# Microbial metabolism disrupts cytokine activity to impact host immune response

**DOI:** 10.1073/pnas.2405719121

**Published:** 2024-11-08

**Authors:** Eleanor K. P. Marshall, Catarina Nunes, Sophie Burbaud, Crystal M. Vincent, Natalie O. Munroe, Carolina J. Simoes da Silva, Ashima Wadhawan, William H. Pearson, Jasper Sangen, Lucas Boeck, R. Andres Floto, Marc S. Dionne

**Affiliations:** ^a^Centre for Bacterial Resistance Biology, Imperial College London, London SW7 2AZ, United Kingdom; ^b^Department of Life Sciences, Imperial College London, London SW7 2AZ, United Kingdom; ^c^Department of Medicine, Molecular Immunity Unit, University of Cambridge, Medical Research Council Laboratory of Molecular Biology, Cambridge CB2 0QH, United Kingdom; ^d^Cambridge Centre for Artificial Intelligence in Medicine, Cambridge CB3 0WA, United Kingdom; ^e^Department of Biomedicine, University of Basel, Basel 4031, Switzerland; ^f^Cambridge Centre for Lung Infection, Royal Papworth Hospital, Cambridge CB2 0AY, United Kingdom

**Keywords:** *Mycobacterium abscessus*, *Drosophila melanogaster*, infection, innate immunity, metabolism

## Abstract

Host–pathogen interactions are shaped by the metabolic status of both the host and pathogen. The host must regulate metabolism to fuel the immune response, while the pathogen must extract metabolic resources from the host to enable its own survival. In this study, we focus on the metabolic interactions of *Mycobacterium abscessus* with *Drosophila melanogaster*. We identify MAB_1132c as an asparagine transporter required for pathogenicity in *M. abscessus*. We show that this requirement is specifically associated with damage to the host: flies infected with MAB_1132c knockout bacteria, or with wild-type bacteria grown in asparagine-restricted conditions, are longer lived without showing a significant change in bacterial load. This is associated with a reduction in the host innate immune response, demonstrated by the decreased transcription of antimicrobial peptides as well as a significant reduction in the ability of the infection to disrupt systemic insulin signaling. Much of the increase in host survival during infection with asparagine-limited *M. abscessus* can be attributed to alterations in unpaired cytokine signaling. This demonstrates that asparagine transport in *M. abscessus* prior to infection is not required for replicative fitness in vivo but does significantly influence the interaction with the host immune responses.

## Significance

*Mycobacterium abscessus* causes severe pulmonary infections in cystic fibrosis patients, and globally infection rates are increasing. As this opportunistic pathogen is typically acquired from environmental sources, it is important to understand how the metabolic status of *M. abscessus* prior to infection can impact host immune responses and infection outcomes. Using a *Drosophila melanogaster* model of *M. abscessus* infection, we have described how the levels of asparagine available to *M. abscessus* prior to an infection lead to changes in innate immune signaling. We demonstrate that by reducing asparagine transport in *M. abscessus*, infected flies live longer without a significant corresponding decrease in bacterial loads and that this effect is driven, in part, by host cytokine signaling.

*Mycobacterium abscessus* has emerged over recent decades as a dangerous pathogen, especially for individuals with underlying lung conditions such as cystic fibrosis and bronchiectasis (1, 2). Globally, there has been an increase in the rates of infection, in part driven by expansions of dominant circulating clones (3–5). As an opportunistic environmental pathogen, *M. abscessus* infection can be acquired from various environmental sources such as contaminated water distribution and plumbing systems (6). Additionally, person-to-person transmission can occur via fomites, long-lived aerosols, and environmental intermediates. This has fueled the spread of *M. abscessus*, creating global transmission networks (3, 7, 8), further compounded by changing climates (9). Infections with *M. abscessus* are extremely challenging to treat due to high levels of both intrinsic and acquired resistance mechanisms, such as the highly impermeable mycomembrane, relatively slow growth rate, and array of antimicrobial drug exporters (10, 11).

Metabolic adaptations of mycobacteria are central to their virulence and antibiotic resistance profiles (12). For mycobacteria, as intracellular pathogens often located within the harsh, nitrogen-limited environment of a phagosome during infection, acquiring nitrogen and other nutrients represents an important facet of intracellular survival. In *Mycobacterium tuberculosis*, uptake of amino acids asparagine and aspartate and the subsequent nitrogen assimilation play an important role in intracellular survival by increasing resistance to acid stress within the phagosome (13, 14). In this organism, nitrogen limitation leads to widespread metabolic reprofiling, often driven by transcriptional changes under the regulation of GlnR (15, 16), and is associated with important clinical phenotypes such as dormancy (17), formation of granulomas (17), biofilm development (18) and antibiotic tolerance (19). Moreover, nitrogen limitation in *M. abscessus* leads to increased triacylglycerol intracellular lipid inclusions within the bacteria, elevated drug tolerance, and increased virulence in zebrafish embryos (19). However, relatively little is known about the specific metabolic adaptations of *M. abscessus*. Due to the profound differences in growth rate between slow- and fast-growing mycobacteria, it is likely that the metabolic strategies of *M. abscessus* are considerably different from those of other pathogenic mycobacteria. An example of such differences between these pathogenic mycobacterial species has recently been described in the context of nitrate metabolism during hypoxia (20).

Mycobacterial infection also leads to changes in the metabolism of the host, which in turn changes the abundance of key metabolites available to the infecting *Mycobacterium*. For mycobacteria within phagocytes, the best-studied of these changes is increased triglyceride deposition within infected macrophages; this dysregulation of intracellular lipid bodies is driven, in part, by cytokine signaling (21–23). The availability of this triglyceride generally benefits the microbe (24–26). On a systemic level, mycobacterial infection generally causes disruptions in insulin signaling, leading to hyperglycemia and wasting of metabolic stores (27–29). These effects have been observed in humans and other mammals but also in invertebrate hosts such as *Drosophila melanogaster*, despite many differences among these hosts in their recognition of mycobacteria and the effector mechanisms they engage (27, 30, 31).

*D. melanogaster* has proven itself to be a useful model organism for the study of mycobacterial host–pathogen interactions, primarily in studying the role of innate immunity in controlling mycobacterial infections. Recently, Touré *et al.* found that infecting flies with mutations in various aspects of either Toll or Imd signaling had no impact on survival during in vivo *M. abscessus* infection (32). Differences in survival did emerge; however, once internalization of *M. abscessus* by host phagocytes was inhibited, shedding light on the role of phagocytes during *M. abscessus* infection and the differences in host responses to intracellular or extracellular bacteria (32). It has also been shown that intracellular *M. abscessus* can resist host cytotoxic responses and survive cell lysis during in vivo infection of *D. melanogaster* as well as in mammalian host cells, a trait shared with other pathogenic mycobacteria (33).

The metabolic adaptations of the host and pathogen during infection are both important determinants of infection outcome. However, the interactions between host metabolism and microbial metabolism are, in general, not well understood: In many cases, it is unclear which host metabolites are most important for microbes and how these metabolites specifically contribute to microbial survival, and whether microbial exploitation of host metabolites interacts with host metabolic regulation or has direct consequences for the host. The relationship between the metabolic adaptations of *M. abscessus* and host immune responses is particularly poorly understood, and given the environmental infection routes of *M. abscessus*, studying these interactions may inform infection control and novel therapeutic strategies.

In this study, we combine microbial and host genetics to probe the immune and metabolic consequences of interactions between *D. melanogaster* and *M. abscessus*. We show that MAB_1132c functions as an asparagine transporter and promotes *M. abscessus* pathogenicity: MAB_1132c knockout bacteria take significantly longer to kill the host despite the absence of any detectable effect on bacterial growth in vivo. This effect is directly attributable to reduced asparagine availability as wild-type bacteria grown in asparagine-limited media exhibit similarly reduced pathogenicity. Remarkably, asparagine transport via MAB_1132c appears to enhance immune activation by the bacterium: Despite being present in identical numbers, MAB_1132c knockout strains drive lower levels of expression of NF-*κ*B target genes than wild-type bacteria. MAB_1132c-dependent pathogenicity and disruptions in insulin signaling are both dependent on signaling via interleukin-like cytokines, *unpaired 2* (*upd2*) and *unpaired 3* (*upd3*). Thus, our work shows how the metabolic status of *M. abscessus* both prior to and during an in vivo infection leads to changes in innate immune activation, which in turn leads to changes in cytokine and insulin signaling, and, ultimately, changes in pathogenicity.

## Results

### MAB_1132c Functions as an Asparagine Permease in *M. abscessus*.

Our previous phenogenomic analysis of *M. abscessus* clinical isolates revealed an association between apparent loss-of-function alleles of MAB_1132c and pathogenicity in *D. melanogaster* (2). The alleles identified were present only in a few clinical isolates from the *M. abscessus massiliense* clade. In order to directly study the role of MAB_1132c in *M. abscessus* pathogenesis, we used a CRISPR-Cas9 system to produce frameshift mutations within the MAB_1132c gene on the ATCC 19977 genetic background. This produced three independent ∆MAB_1132c strains, as described in *SI Appendix*, Table S1. Most experiments were performed using all three ∆MAB_1132c strains; their behavior was essentially identical.

**Fig. 1.**
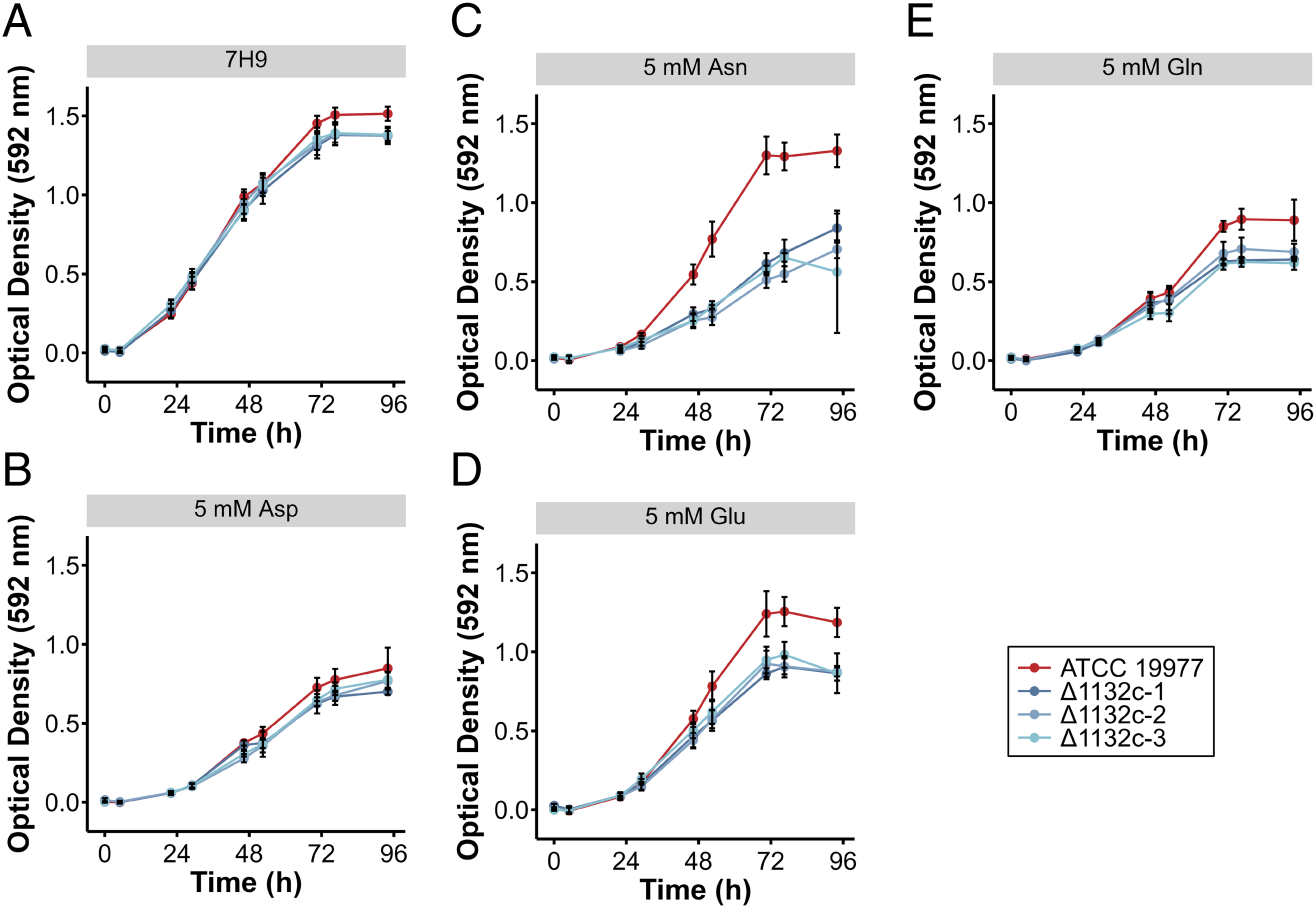
MAB_1132c is important but not essential for growth with asparagine as the sole nitrogen source. Growth of *M. abscessus* ATCC 19977 (red) and ∆MAB_1132c strains (blue) in various nutrient conditions, measured by optical density at 592 nm. Data represent the mean ± SD of quadruplicate samples and are representative of three independent experiments. Bacteria grown in (*A*) complete 7H9 media, or minimal media containing 5 mM of (*B*) aspartate (Asp), (*C*) asparagine (Asn), (*D*) glutamate (Glu), or (*E*) glutamine (Gln) as the sole nitrogen source.

MAB_1132c encodes one of two predicted L-asparagine permeases in *M. abscessus*. However, in *M. tuberculosis*, one of two predicted L-asparagine permeases apparently functions as an aspartate transporter (13, 14). We therefore tested whether MAB_1132c had a role in L-asparagine transport by evaluating the growth of ∆MAB_1132c strains in complete medium (7H9) (Fig. 1*A*) and minimal medium containing 5 mM of one of the structurally related amino acids, aspartate (Fig. 1*B*), asparagine (Fig. 1*C*), glutamate (Fig. 1*D*), or glutamine (Fig. 1*E*), as the sole nitrogen source. In complete medium and in minimal medium containing aspartate as the sole nitrogen source, ∆MAB_1132c strains exhibited no growth defect relative to the parental strain (Fig. 1*B*). However, ∆MAB_1132c strains did show a mild growth defect in media containing only glutamate (Fig. 1*D*) or glutamine (Fig. 1*E*) and a strong growth defect in media containing only asparagine as a nitrogen source (Fig. 1*C*). This requirement suggests that MAB_1132c functions as an asparagine transporter in *M. abscessus*, with possible roles in transport of glutamine and glutamate; the fact that ∆MAB_1132c bacteria are not completely incapable of growth in media with asparagine as the sole nitrogen source clearly indicates that MAB_1132c is not the only transport system for this amino acid. This was confirmed by growing the ∆MAB_1132c strains in minimal media with increasing concentrations of asparagine as the sole nitrogen source (5 mM to 40 mM) (*SI Appendix*, Fig. S1 *A*–*D*). While growth of the wild-type strain was not particularly impacted by increasing asparagine availability, ∆MAB_1132c strains showed increasing ability to grow correlating with the concentration of asparagine, reaching growth rates similar to the parental strain at 40 mM asparagine. Transcript levels of the other predicted L-asparagine permease, MAB_2914, were similar between wild-type *M. abscessus* and ∆MAB_1132c strains during exponential phase growth in either complete media (*SI Appendix*, Fig. S1*E*) or minimal media with 5 mM asparagine as the sole nitrogen source (*SI Appendix*, Fig. S1*F*). We conclude that MAB_1132c functions as one of several asparagine transporters in *M. abscessus* which function nonredundantly, highlighting the importance of asparagine transport in this bacterium.

### Asparagine Acquisition by *M. abscessus* Impacts Bacterial Virulence and Host Tolerance.

**Fig. 2.**
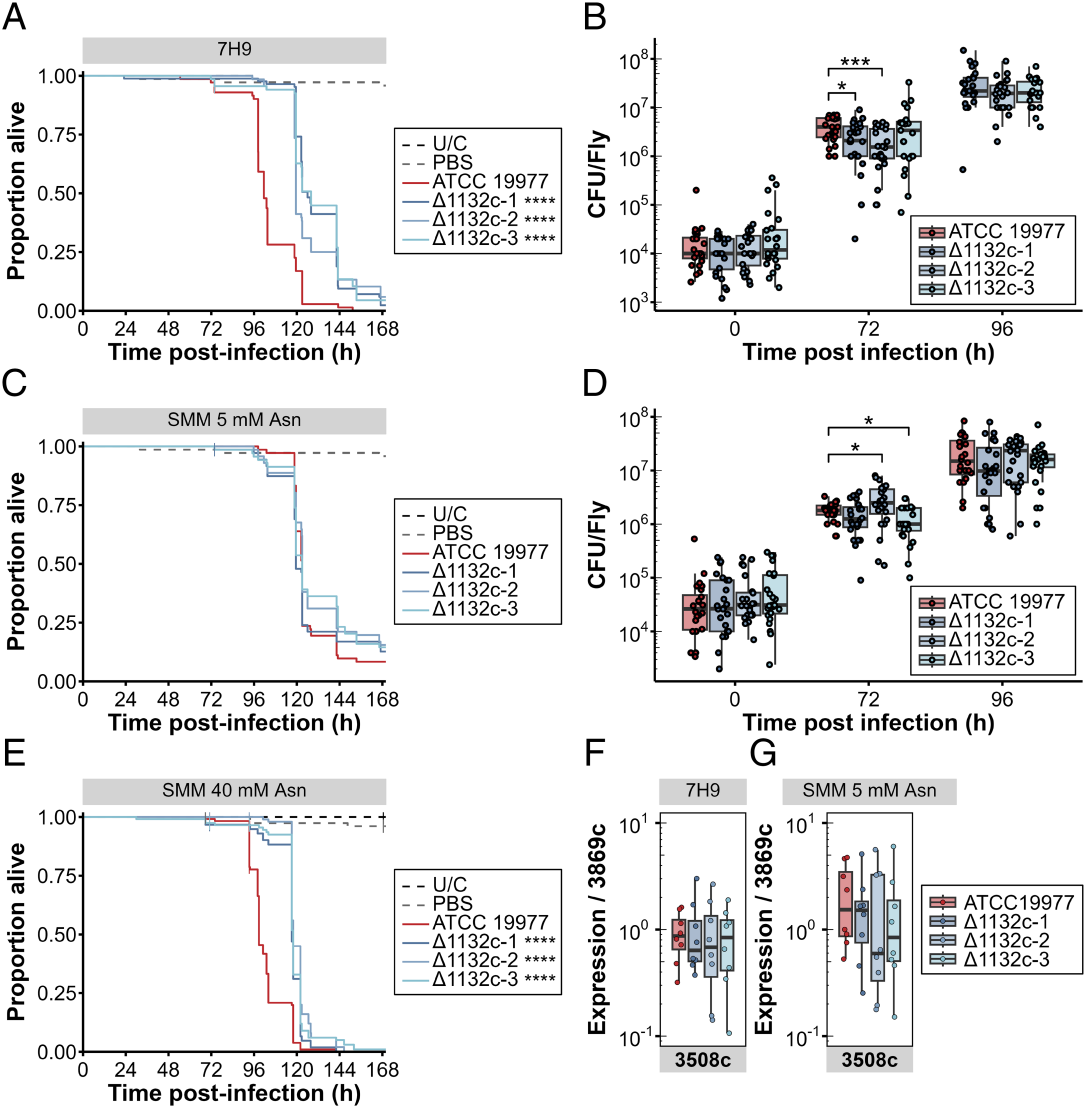
Asparagine availability in *M. abscessus* impacts host survival but not bacterial in vivo growth. (*A*) Survival and (*B*) bacterial loads of *w*^1118^ flies infected with *M. abscessus* ATCC 19977 or ∆MAB_1132c strains grown in complete medium (7H9). (*C*) Survival and (*D*) bacterial loads of *w*^1118^ flies infected with *M. abscessus* ATCC 19977 or ∆MAB_1132c strains grown in Sauton’s minimal medium (SMM) with 5 mM asparagine as the sole nitrogen source. (*E*) Survival of *w*^1118^ flies infected with *M. abscessus* ATCC 19977 or ∆MAB_1132c strains grown in Sauton’s minimal media (SMM) with 40 mM asparagine as the sole nitrogen source. (*F*, *G*) Expression of transcriptional regulator *whiB7* (MAB_3508c) normalised against housekeeping gene *rpoB* (MAB_3869c) in *M. abscessus* ATCC 19977 and ∆MAB_1132c strains following growth to log phase in (*F*) complete medium (7H9) or (*G*) SMM with 5 mM asparagine. Survival data are pooled from three independent experiments performed with common controls, 123 ≥ n ≥ 68 per group. Asterisks in the legend indicate the *P*-value compared to flies infected with ATCC 19977, compared using the log-rank test. Bacterial load data are pooled from three independent experiments, n = 8 per strain per condition per independent experiment. Statistical significance was calculated by comparing the mutant strains to ATCC 19977 within the same time point and media using Kruskal–Wallis ANOVAs. Bacterial gene expression data are representative of two independent experiments (n = 8 per strain), and were compared using a Kruskal-Wallis ANOVA. ^∗^*P* < 0.05; ^∗∗^*P* < 0.01; ^∗∗∗^*P* < 0.001; ^∗∗∗∗^*P* < 0.0001.

Our previous phenogenomic analysis indicated that MAB_1132c had some effect on host interaction, but the nature of this effect was unclear. Infection of flies with clinical isolates of *M. abscessus* containing loss-of-function alleles in MAB_1132c led to prolonged survival of infected *D. melanogaster*, relative to infection with wild-type *M. abscessus* (2). To determine how asparagine deprivation would impact *M. abscessus* virulence, we assayed the survival of flies infected with wild-type or ∆MAB_1132c bacteria. ∆MAB_1132c *M. abscessus* cultured in complete 7H9 medium consistently killed flies one day later than wild-type *M. abscessus* (Fig. 2*A*); however, there was minimal difference in bacterial loads (Fig. 2*B*) at 72 h postinfection between the wild-type and ∆MAB_1132c strains. We then assessed fly survival and bacterial load following culture of *M. abscessus* in minimal medium supplemented with low (5 mM) asparagine as the sole nitrogen source. Growth in low-asparagine medium caused the wild-type strain to recapitulate the behavior of ∆MAB_1132c strains: Host death was delayed (Fig. 2*C*) without any change in the numbers of viable *M. abscessus* present at any point in infection (Fig. 2*D*). This was consistent with our observations of bacteria grown in complete 7H9 medium, that wild-type and ∆MAB_1132c mutant bacteria proliferated at similar rates in the fly independent of their preinfection growth conditions. Increasing asparagine availability in the minimal medium to 40 mM restored the wild-type *M. abscessus* phenotype to that seen in initial growth in complete 7H9 medium (Fig. 2*E*). This indicates that asparagine availability prior to infection enhances bacterial pathogenicity without altering in vivo proliferative ability.

Previous work showed that nitrogen-limited *M. abscessus* showed accumulation of intracellular triacylglycerol stores (19). We reproduced the observation of increased triacylglycerol in wild-type bacteria grown in low nitrogen minimal medium (0.05 g/L ammonium chloride) compared to high nitrogen minimal medium (1 g/L ammonium chloride) (*SI Appendix*, Fig. S2*A*). No triacylglycerol accumulation was observed in ∆MAB_1132c bacteria grown in complete 7H9 media (*SI Appendix*, Fig. S2*B*). Growth of the bacterial strains in minimal medium with 5 mM asparagine as the sole nitrogen source (*SI Appendix*, Fig. S2*C*) did result in triacylglycerol accumulation in the ∆MAB_1132c strains, suggesting that the impaired in vitro growth of these strains in this medium was due to nitrogen limitation. However, we observed considerably less triacylglycerol accumulation in wild-type bacteria grown in low asparagine media relative to the ∆MAB_1132c strains. The conditions that exhibit markedly delayed host death, infection with the ∆MAB_1132c bacteria grown in any medium or wild-type bacteria grown in low-asparagine media, do not show consistent changes in triacylglycerol levels. Additionally, infection of *w*^1118^ flies with wild-type *M. abscessus* grown in minimal media containing either high nitrogen concentrations or low nitrogen concentrations shows no clear difference in host survival, unlike the previous observations in infection of zebrafish embryos (19) (*SI Appendix*, Fig. S2*D*). We therefore conclude that decreased virulence associated with decreased asparagine uptake, either due to MAB_1132c mutation or growth in low-asparagine media, is not a product of general nitrogen limitation. Instead, our results suggest that the decrease in virulence stems from a specific requirement for asparagine transport prior to and during infection.

*M. abscessus* is capable of producing a thick layer of glycopeptidolipids surrounding the mycomembrane; this can result in highly variable host immune responses based on the presence or absence of these glycopeptidolipids due to differences in cord formation and exposed immunogenic proteins (34–36). We reasoned that the virulence differences observed might result from differences in glycopeptidolipid production. The presence of the glycopeptidolipid layer causes the bacteria to adopt a smooth morphotype, while *M. abscessus* lacking glycopeptidolipids adopts a rough morphotype. We observed no visible morphotype difference between these bacteria strains. Moreover, we measured transcript levels of 13 key genes required for production and transport of glycopeptidolipids. We found no difference in the expression of these genes between wild-type bacteria and the ∆MAB_1132c strains grown in complete 7H9 medium (*SI Appendix*, Fig. S3*A*) or low-asparagine minimal medium (*SI Appendix*, Fig. S3*B*). In addition to the 13 GPL genes, we also measured transcription of *whiB7* (MAB_3508c), which is involved in the transcriptional regulation of various *M. abscessus* virulence factors (Fig. 2 *F*–*G*). This also showed no difference between wild-type bacteria and the ∆MAB_1132c strains in either medium.

### Changes in Host Innate Immune Signaling Based on *M. abscessus* Asparagine Uptake.

The fact that ∆MAB_1132c bacteria caused significantly delayed host death despite being present in similar numbers indicated that their interaction with the host was fundamentally different. In order to get a better understanding of the nature of this difference, we assayed the expression of antimicrobial peptides, well-defined transcriptional targets of innate immune detection pathways, after infection with wild-type and ∆MAB_1132c *M. abscessus*.

**Fig. 3.**
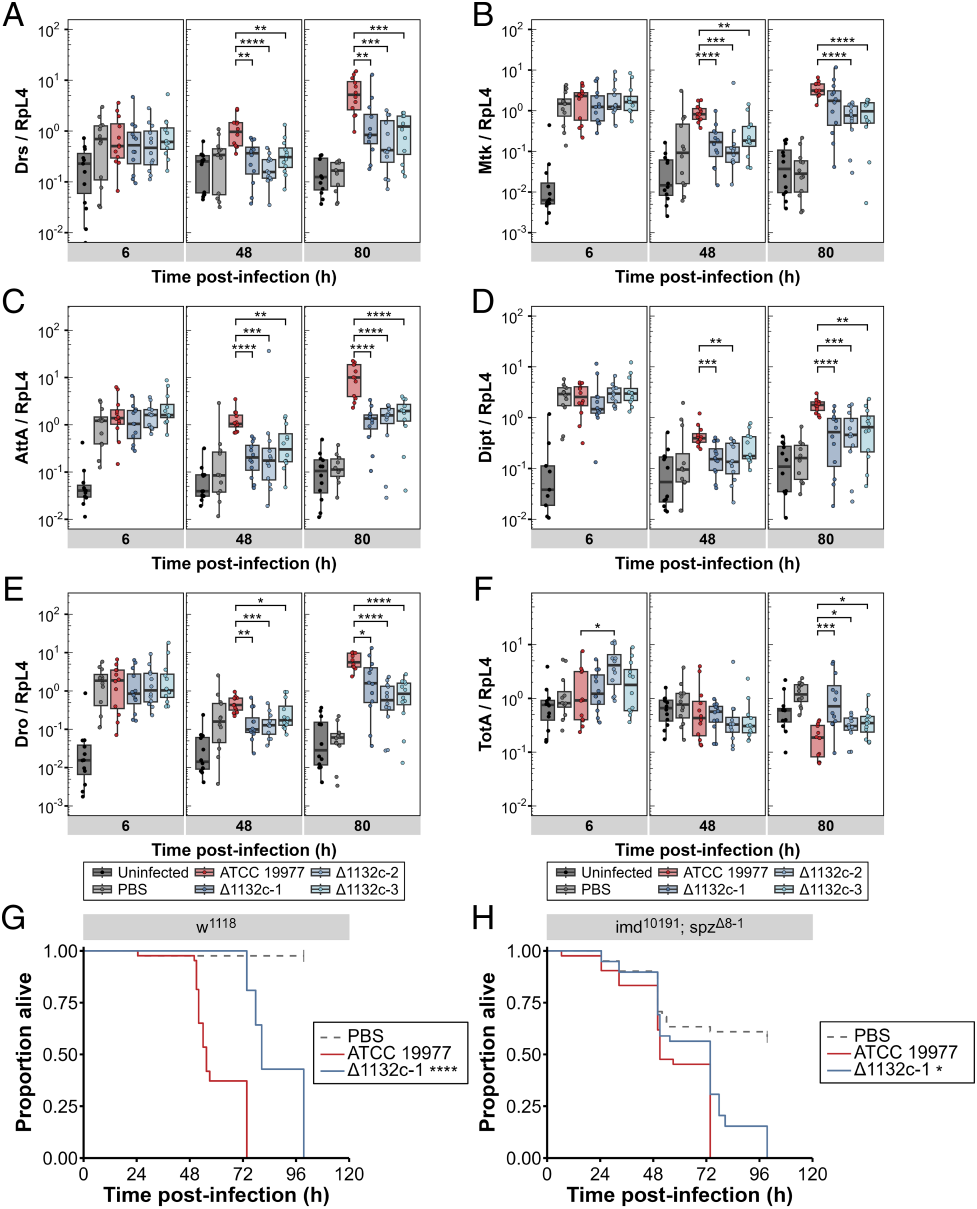
Reduction in antimicrobial peptide transcription by ∆MAB_1132c *M. abscessus*. RT-qPCRs show transcription of antimicrobial peptides in flies infected with *M. abscessus* ATCC 19977 (red) or ∆MAB_1132c strains (blue) over the course of an infection. Uninjected (black) and PBS Tween80 0.1% sterile-injury (gray) flies are included as controls. Antimicrobial peptide transcript levels shown are (*A*) *Drosomycin*, (*B*) *Metchnikowin*, (*C*) *Attacin A*, (*D*) *Diptericin*, and (*E*) *Drosocin*. Stress protein (*F*) *Turandot A* is also shown. Data are representative of three independent experiments (N = 12), normalized against *RpL4*, and were compared using a Kruskal–Wallis ANOVA. Statistical comparisons between *M. abscessus* ATCC 19977 and the ∆MAB_1132c strains are shown, all statistically significant comparisons are available in *SI Appendix*, Table S7. Survival curves of (*G*) *w*^1118^ and (*H*) *imd*^10191^*; spz*^∆8−1^ flies infected with *M. abscessus* ATCC 19977 (red) or ∆MAB_1132c-1 (blue), grown in complete 7H9 media. The control group is PBS Tween80 0.1% injected flies (gray). Data represent a single experiment, with at least 40 flies per group. Asterisks in the legend indicate the *P*-value compared to flies infected with ATCC 19977, compared using the log-rank test. ^∗^*P* < 0.05; ^∗∗^*P* < 0.01; ^∗∗∗^*P* < 0.001; ^∗∗∗∗^*P* < 0.0001.

We consistently observed that *M. abscessus*, like other mycobacteria, drives slower and weaker immune activation than most other tested bacteria in *D. melanogaster* (30). Infection does not activate antimicrobial peptide expression to a greater extent than the sterile-injury PBS control within the first 6 h postinfection (Fig. 3 *A*–*E*). However, by 48 h postinfection, antimicrobial peptide transcript levels are significantly higher in flies infected with wild-type *M. abscessus* than in PBS controls, an effect that is strengthened at 80 h postinfection. This late antimicrobial peptide induction is significantly blunted in flies infected with ∆MAB_1132c bacteria; it is not until 80 h postinfection that antimicrobial peptide transcription in flies infected with ∆MAB_1132c exceeds that seen in the PBS sterile-injury control group. This pattern is true of antimicrobial peptides associated with both Toll and Imd pathway activation, as well as the antimicrobial peptide-like Bomanins *BomS1* and *BomS2* (*SI Appendix*, Fig. S4 *A* and *B*). Together, these observations clearly indicate that ∆MAB_1132c bacteria are significantly less active in driving activation of Toll and Imd pathways, despite being present at similar numbers to wild-type bacteria.

Antimicrobial peptides are the best-known transcriptional readouts of immune activation in *D. melanogaster*, but other immune target genes are also relevant to survival following infection. Stress protein *Turandot A* (*TotA*) helps protect the host from inflammatory damage and is induced in the fat body upon JAK/STAT signaling by the cytokines *unpaired (upd)2* and *upd3* (37–39). At 80 h postinfection, *TotA* expression was mildly increased in PBS sterile-injury flies and reduced in wild-type *M. abscessus* infected flies, relative to the uninfected controls. Consistent with the blunted transcriptional responses of antimicrobial peptides in flies infected with ∆MAB_1132c strains, the expression of *TotA* in these flies was reduced compared to the PBS sterile-injury flies, but not as low as the wild-type *M. abscessus* infected flies (Fig. 3*F*). While this suggests that there is increased *upd2* or *upd3* activity driving JAK/STAT signaling, we could not detect a change in *upd2* or *upd3* mRNA expression (*SI Appendix*, Fig. S4 *C* and *D*).

To confirm the importance of the observed decrease in immune signaling activation during infection with ∆MAB_1132c strains, we infected flies with mutations in both *imd* and *spz*, eliminating both Imd and Toll pathway signaling. These *imd*^10191^*; spz*^∆8−1^ flies exhibited a reduction in survival time after *M. abscessus* infection compared with *w*^1118^ controls (Fig. 3 *G* and *H*). However, the difference between *M. abscessus* ATCC 19977 and ∆MAB_1132c-1 is almost entirely lost in *imd*^10191^*; spz*^∆8−1^ flies (Fig. 3 *G* and *H*). The change in survival of ∆MAB_1132c-infected flies, combined with the decreased transcriptional response, indicates a clear change in the host–pathogen interactions at play, including the balance between changes in the susceptibility of ∆MAB_1132c to host AMPs and the extent of self-inflicted damage to host tissues by the inflammatory response to the infection.

### *D. melanogaster* Cytokines are Responsible for Changes in Host Susceptibility to Asparagine-Limited *M. abscessus*.

Immune-induced cytokine signaling is an important cause of pathology in many infections. We had previously observed that the cytokine *upd3* was an important regulator of the *D. melanogaster* immune response to *Mycobacterium marinum,* whereby loss of *upd3* function prolonged fly survival (21). The reduced immune activation and elevated *TotA* expression we observed with MAB_1132c knockout bacteria suggested the possibility that differences in time to death might be a product of changes in cytokine activity, so we tested the role of *upd3* and its relative *upd2* in infections with wild-type and ∆MAB_1132c *M. abscessus*.

**Fig. 4.**
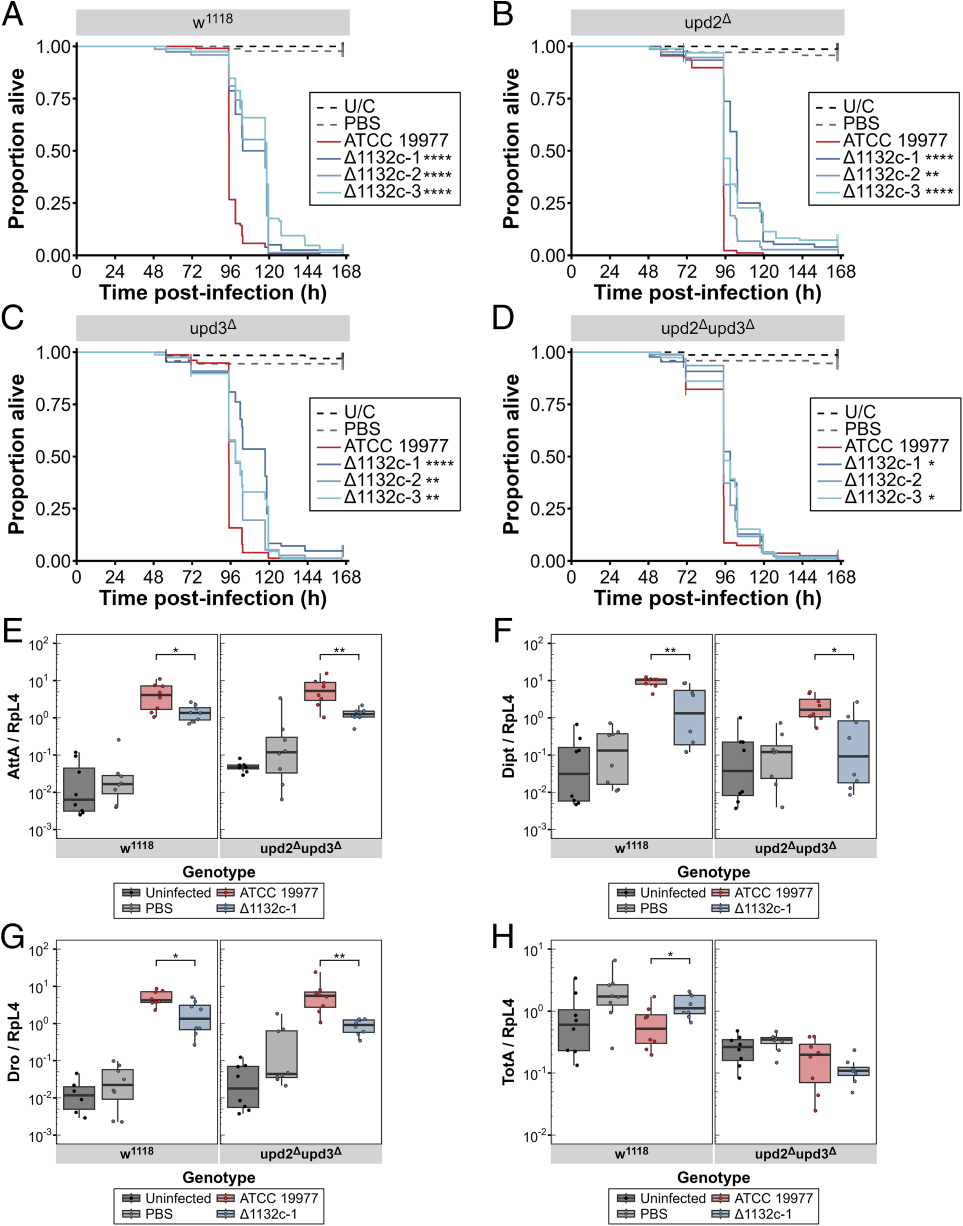
Increased susceptibility of unpaired mutant *D. melanogaster* to asparagine-limited *M. abscessus*. Survival curves of (*A*) *w*^1118^ (control), (*B*) *upd2*^∆^, (*C*) *upd3*^∆^, or (*D*) *upd2*^∆^*upd3*^∆^ flies infected with *M. abscessus* ATCC 19977 (red) or ∆MAB_1132c strains (blue) grown in complete media (7H9). Control groups are uninfected flies (black) and PBS Tween80 0.1% injected flies (gray). Data represent three independent experiments with a minimum of 20 flies per group per experiment and were analyzed using the log-rank test. Asterisks in legends indicate significant differences compared against ATCC 19977. RT-qPCRs show transcription of antimicrobial peptides in *w*^1118^ and *upd2*^∆^*upd3*^∆^ flies infected with *M. abscessus* ATCC 19977 (red) or ∆MAB_1132c-1 (blue) at 80 h postinfection. Uninjected (black) and PBS Tween80 0.1% sterile-injury (gray) flies are included as controls. Transcript levels shown are (*E*) *Attacin A*, (*F*) *Diptericin*, (*G*) *Drosocin*, and (*H*) *Turandot A*. Data are representative of two independent experiments (N = 8), normalized against *RpL4*, and were compared using a Kruskal–Wallis ANOVA. ^∗^*P* < 0.05; ^∗∗^*P* < 0.01; ^∗∗∗^*P* < 0.001; ^∗∗∗∗^*P* < 0.0001.

We observed that wild-type *M. abscessus* did not show the same effect as *M. marinum*: Flies lacking either *upd2* or *upd3*, or both cytokines, exhibited identical time-to-death as controls. However, the difference in time-to-death between flies infected with wild-type and ∆MAB_1132c *M. abscessus* was significantly reduced in flies carrying mutations in *upd2* (Fig. 4*B* or *upd3* (Fig. 4*C*), and almost completely eliminated in *upd2 upd3* double mutants (Fig. 4*D*), relative to wild-type flies (Fig. 4*A*). This suggests that asparagine acquisition via MAB_1132c in this infection ultimately contributes to inhibition of the activity of *upd2* and *upd3*; genetic elimination of these cytokines is sufficient to almost entirely revert the ∆MAB_1132c survival phenotype to that of wild-type *M. abscessus*.

We next tested whether the action of *upd2* and *upd3* that was prolonging survival of flies infected with ∆MAB_1132c was also responsible for the reduction in antimicrobial peptide production observed at late time points of this infection. While *D. melanogaster* JAK/STAT signaling is not directly involved in antimicrobial peptide transcription, there is a degree of cross-talk across the various immune signaling pathways. The reduction in antimicrobial peptide transcription of flies infected with ∆MAB_1132c relative to flies infected with wild-type *M. abscessus* was still observed in *upd2 upd3* double mutant flies, including *Attacin A* (Fig. 4*E*), *Diptericin* (Fig. 4*F*), and *Drosocin* (Fig. 4*G*). *TotA* expression (Fig. 4*H*) was reduced across all groups in *upd2 upd3* flies compared with w^1118^ flies, but notably the increase in *TotA* expression seen in w^1118^ flies infected with ∆MAB_1132c was abrogated in the *upd2 upd3* flies. This indicates that while *upd2* and *upd3* are responsible for the differences in *TotA* expression and host survival between wild-type *M. abscessus* and ∆MAB_1132c, it is not involved in the changes to host antimicrobial peptide transcription. We also confirmed that the shift in survival in *upd* mutant flies is not a product of general nitrogen limitation by infecting with *M. abscessus* ATCC 19977 grown in high- and low-nitrogen media (*SI Appendix*, Fig. S2 *E*–*G*).

It is unclear what change is occurring in *M. abscessus* under asparagine-limited to drive this interaction with host cytokine activity; however, as transcript levels of *upd2* (*SI Appendix*, Fig. S4*C*), *upd3* (*SI Appendix*, Fig. S4*D*), and *Socs36E* (*SI Appendix*, Fig. S4*E*), the negative regulator of JAK/STAT signaling downstream of *upd2* and *upd3*, are unchanged across all groups, it is likely that this interaction is posttranslational. Additionally, there was no change across infection conditions in the transcript levels of the TNF*α* homolog *egr* (*SI Appendix*, Fig. S4*F*) and its processing protease *Tace* (*SI Appendix*, Fig. S4*G*). egr drives JNK signaling in *D. melanogaster*, which can up-regulate transcription of upd ligands (40). Further, there was no change in *Atg2* expression (*SI Appendix*, Fig. S4*H*), which was previously shown to be down-regulated by JAK/STAT signaling via *upd3* during *M. marinum* infection (21).

### Asparagine Uptake by *M. abscessus* Drives Changes in Host Insulin/AKT Signaling.

Mycobacterial infections drive insulin signaling disruption in humans and *D. melanogaster*, and we have previously shown that increased activity of the insulin/AKT-inhibited transcriptional factor *FoxO* is an important driver of pathology in *D. melanogaster* infected with *M. marinum* (27). upd cytokine signaling is an important regulator of insulin/AKT pathway activity in *D. melanogaster* via effects on insulin-like peptide production, release, and peripheral sensitivity (41–43). Therefore, we next tested the effects of MAB_1132c on AKT activity by assaying levels of AKT phosphorylation of Ser505 (27, 44).

**Fig. 5.**
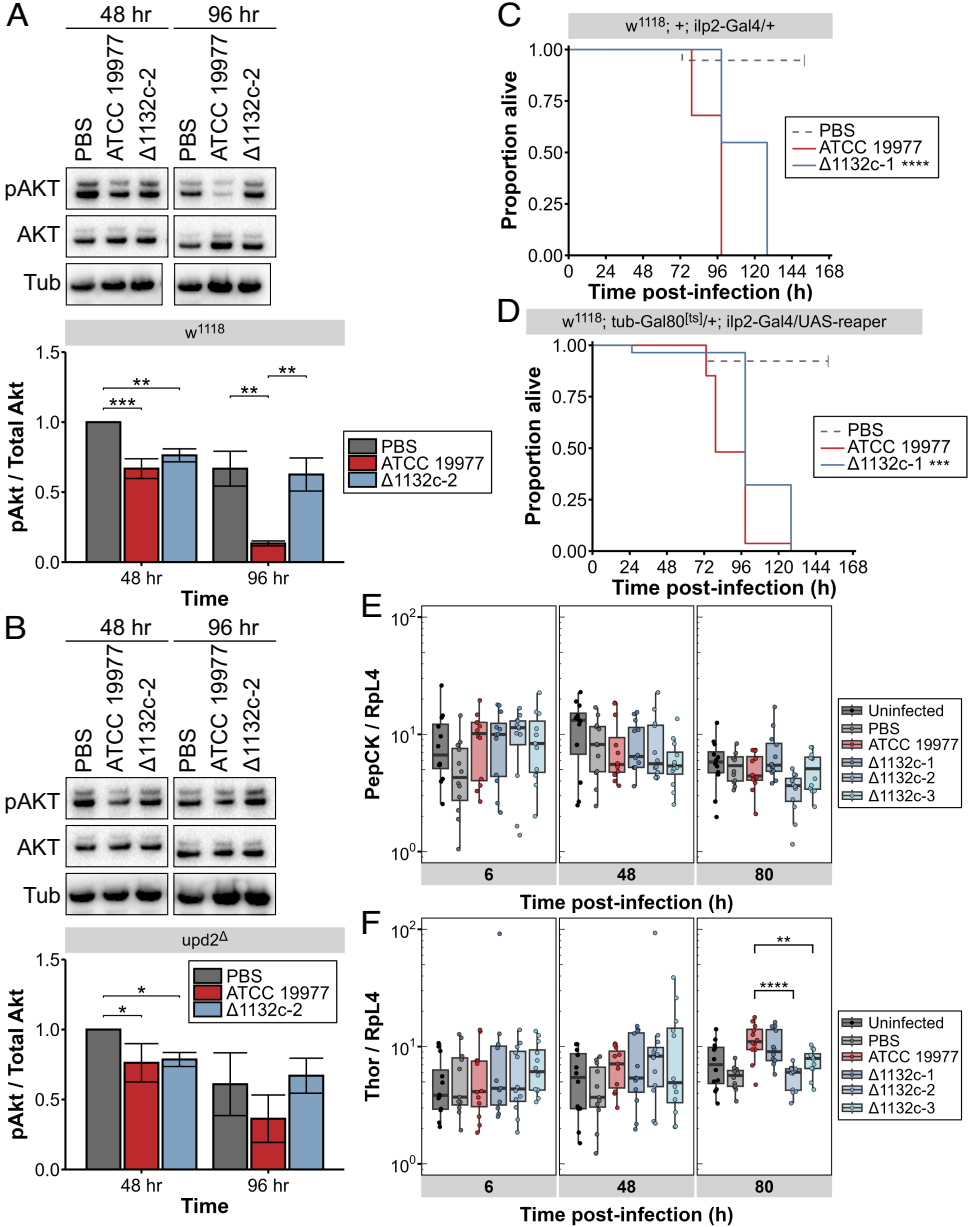
Reduction of activated AKT in *D. melanogaster* by *M. abscessus* is partially dependent on bacterial asparagine uptake and host *upd2* signaling. Systemic levels of phosphorylated Ser505 (pAKT) and total AKT protein of (*A*) *w*^1118^ and (*B*) *upd2*^∆^ flies at 48 and 96 h postinfection with *M. abscessus* ATCC 19977 (red) or ∆MAB_1132c-2 (blue) cultured in complete 7H9 media, measured by western blot. The control group is PBS Tween80 0.1% injected flies (gray). Data represent one independent experiment; n = 3 per strain per genotype. Groups were compared with one-way ANOVA with Tukey’s test. Survival of (*C*) *w*^1118^; +; ilp2-Gal4/+ (control) and (*D*) *w*^1118^; tub-Gal80^[^*^ts^*^]^/+; ilp2-Gal4/UAS-reaper (IPC-depleted) flies infected with *M. abscessus* ATCC 19977 (red) or ∆MAB_1132c-1 (blue). Control groups are PBS Tween80 0.1% injected flies (gray). Data represent one experiment, with at least 20 flies per group. Asterisks in the legend indicate the *P*-value compared to flies infected with ATCC 19977, compared using the log-rank test. RT-qPCRs show transcript levels of (*E*) *PepCK* and (*F*) *Thor* in *w*^1118^ flies infected with *M. abscessus* ATCC 19977 (red) or ∆MAB_1132c strains (blue) over the course of an infection. Uninjected (black) and PBS Tween80 0.1% sterile-injury (gray) flies are included as controls. Data are representative of three independent experiments (N = 12), normalized against *RpL4*, and were compared using a Kruskal–Wallis ANOVA. Statistical comparisons between *M. abscessus* ATCC 19977 and the ∆MAB_1132c strains are shown. ^∗^*P* < 0.05; ^∗∗^*P* < 0.01; ^∗∗∗^*P* < 0.001; ^∗∗∗∗^*P* < 0.0001.

Consistent with our previous observations of *D. melanogaster* infected with *M. marinum* (27), wild-type *M. abscessus* caused a progressive decrease in the levels of the 60 kDa isoform of phospho-AKT with no changes in the amount of total AKT in wild-type flies. In contrast, wild-type flies infected with ∆MAB_1132c showed a slight initial decrease in the levels of phospho-AKT compared with sterile-injury control flies at 48 h postinfection, but by 96 h postinfection, there was no difference between ∆MAB_1132c infected flies and sterile injury controls (Fig. 5*A*). The reduction in phospho-AKT was retained, though somewhat blunted, in *upd2* mutant flies infected with wild-type bacteria, but the behavior of ∆MAB_1132c infection was completely unaltered in *upd2* mutant hosts (Fig. 5*B*).

This suggests that during infection with wild-type *M. abscessus*, as with *M. marinum* infection of *D. melanogaster* (27), impaired insulin signaling may be an important driver of pathology and that this might be the critical difference in pathology between wild-type and ∆MAB_1132c bacteria. In this case, systemic reduction in insulin signaling should render flies equally sensitive to infection with either strain. We used a Gal80^[^*^ts^*^]^ system to induce apoptosis of insulin-producing cells (IPCs) in adult flies prior to infection. Control flies (Fig. 5*C*) showed the same prolonged survival during infection with ∆MAB_1132c-1 relative to infection with ATCC 19977. We found that loss of IPCs (Fig. 5*D*) only slightly reduced the survival of ∆MAB_1132c-1 infected flies and was not enough to restore the wild-type phenotype. Additionally, RT-qPCR experiments were performed to measure transcript levels of *PepCK* (Fig. 5*E*) and *Thor* (*4E-BP*) (Fig. 5*F*), both transcriptional targets of *FoxO* activation. While there was no significant change in *PepCK* transcription at any time during infection between ATCC19977 and ∆MAB_1132c-infected flies, there was a decrease in *Thor* transcription at 80 h postinfection in two of the three ∆MAB_1132c strains relative to ATCC 19977. Increased *Thor* transcription is indicative of repressed insulin signaling, which is consistent with the decreased phospho-AKT levels observed in ATCC 19977-infected flies late during infection, as AKT acts as an inhibitor of *FoxO*.

Overall, these data indicate that the dysregulation in insulin signaling caused by *M. abscessus* requires asparagine uptake by the bacteria. This may be driven in part by changes in signaling via *upd2* and possibly *upd3*. However, as the shift in survival time during infection of *upd2*^∆^*upd3*^∆^ flies with ∆MAB_1132c strains was not replicated in flies lacking IPCs, this difference in host survival is not a product of differential insulin pathway disruption. Nonetheless, the decoupling between bacterial numbers, host survival, and phospho-AKT levels seen in ∆MAB_1132c infection clearly shows that changes in microbial metabolism can have direct impacts on the metabolic pathology seen during infection. These impacts result, at least in part, from changes in molecules detected by the host and the subsequent host response.

## Discussion

MAB_1132c was originally identified from a high-dimensional phenogenomic analysis of *M. abscessus* clinical isolates, with changes in MAB_1132c being associated with differences in pathogenicity in flies and clofazimine resistance (2). The results of our study show the importance of MAB_1132c during infection of *D. melanogaster*, providing insight into how metabolic regulation in *M. abscessus* contributes to its immune evasion strategies by altering host–pathogen interactions. Because of the nature of *M. abscessus* infection from environmental sources, following by intrahost adaptation and further transmission via environmental intermediates (45), understanding how the metabolic adaptations of *M. abscessus* results in changes to interactions with host immune systems is essential.

The findings of this study confirm the role of MAB_1132c as an asparagine transporter; however, like *M. tuberculosis*, *M. abscessus* appears to also possess additional transporters capable of asparagine uptake, albeit not enough to fully compensate for the loss of MAB_1132c in low asparagine conditions. Despite this in vitro growth defect, the reduction in asparagine transport during in vivo infection has no impact on growth of ∆MAB_1132c strains within *D. melanogaster*. This represents an interesting parallel with the situation in *M. tuberculosis*. Here, expression of asparagine transporter AnsP2 (Rv0346c) was strongly induced in the lungs of patients (46). However, an AnsP2-KO *M. tuberculosis* strain exhibited no difference in growth in the lungs and spleen of infected mice compared with wild-type; mouse survival was not reported (14).

Beyond the function of MAB_1132c during *M. abscessus* infection, the results of this study shed light on a more general role of asparagine transport by *M. abscessus* and how this leads to fundamental changes in host–pathogen interactions. Intriguingly, by restricting asparagine in *M. abscessus*, either by mutating MAB_1132c or by using asparagine-limited media, infected *D. melanogaster* were longer lived despite no detectable difference in bacterial growth in vivo. This suggests that while *M. abscessus* is not reliant on prior asparagine uptake for replicative fitness in vivo, there is some changed *M. abscessus* factor responsible for the increase in host survival time. We observed that bacteria lacking MAB_1132c drove significantly reduced host immune activation. This reduction in immune activation resulted in reduced pathogenicity, enabling the host to live longer and infecting bacteria to grow to higher levels. This difference in host survival is driven, at least in part, by *upd* cytokine signaling, with a possible protective effect offered by increased *TotA* expression. This suggests that when *M. abscessus* infects with sufficient asparagine levels, the cytokine signaling driven by upd is normally inhibited or ineffectual.

The differences we observe in interactions with the host may stem from differences in production of one or more *M. abscessus* pathogenic effectors. We have excluded changes in triglyceride accumulation or glycopeptidolipid synthesis as relevant mechanisms, but we have been unable to identify a specific *M. abscessus* factor that is changed by asparagine availability and is responsible for the observed differences in host interaction. Changes in nitrogen metabolism in other pathogenic mycobacteria have been shown to lead to changes in cell wall structure due to GlnR regulation of the poly-L-glutamine (PGL) layer (47). Since the *Drosophila* immune response is dependent on direct sensing of peptidoglycan, changes of this kind could explain the reduction of antimicrobial peptide transcription in the ∆MAB_1132c infected flies despite carrying similar bacterial loads. However, it is unclear whether the poly-L-glutamine layer exists in *M. abscessus*, let alone whether or not it is affected by MAB_1132c mutation.

Relative to other bacterial infections in *D. melanogaster*, the expression of AMPs in response to *M. abscessus* is not particularly high. Nevertheless, the production of AMPs and other inflammatory responses by the host can damage the host itself (39). While it seems unlikely that *D. melanogaster* AMPs have significant direct antimicrobial action against *M. abscessus*, they may drive further inflammatory damage to host tissues, and they are costly to produce. Therefore, the decrease in AMP transcription seen in ∆MAB_1132c infected flies may contribute the prolonged survival of these flies. Additionally, stress response proteins such as *TotA* can protect host tissues from AMP-mediated damage, so the increased *TotA* expression in ∆MAB_1132c infected flies may add an additional layer of protection against host-derived cytotoxicity (39). It is worth noting that *TotA* expression also requires the NF-*κ*B transcription factor Relish within the fat body, which also drives transcription of AMPs in the IMD signaling pathway (37).

In *Drosophila*, as in other animals, immune activation drives changes in systemic metabolism, mediated partly via loss of systemic insulin signaling activity. Launching an effective immune response is energetically costly for the host organism, requiring a systemic shift in energetic stores and metabolic processes to supply the energy and molecular components needed. We observe that *upd2* is partially required for the drastic reduction in phospho-AKT levels seen during *M. abscessus* infection, which leads to repressed insulin signaling. However, unlike in infections with *M. marinum*, we find that impaired insulin signaling does not appear to be an important driver of pathology in flies infected with *M. abscessus*, and the fact that this change is ameliorated in infection with ∆MAB_1132c is not an important driver of its reduced pathogenicity. Further experiments are required to understand the full impact that microbial asparagine metabolism has on the host immune and metabolic responses to the infection.

Another outstanding question is how changing the asparagine levels in *M. abscessus* prior to infection may change the in vivo competition for asparagine. Limited availability of nutrients creates intense competition between host and pathogen for resources, including amino acids which are highly beneficial to both the host defense and for bacterial virulence. *M. tuberculosis* infections cause consistent metabolic changes in infected hosts, conserved across humans, mice, and zebrafish, including a decrease in host asparagine levels during infection (48). It is unknown whether the same asparagine decrease occurs in flies infected with *M. abscessus*, and if so, how the asparagine is being utilized.

In summary, we show how the metabolic status of *M. abscessus* can influence the host immune response, specifically by altering cytokine signaling, AMP transcription, and insulin signaling pathways. This represents a remarkable convergence between microbial and host metabolic regulation, with significant effects on microbial pathogenicity. Our findings show that pathogen nutrient availability can have far-reaching consequences on host defense, providing broad avenues for exploring host–pathogen interactions and the manipulation of host immune responses in the treatment of mycobacterial infections. The key outstanding question from this work is what specific *M. abscessus* factor is altered by asparagine availability, and subsequently what causes this change in interaction with the host immune systems. In-depth transcriptomic or proteomic studies may shed

further light on this.

## Methods

### Bacterial Strains and Culture Conditions.

*M. abscessus* strains were cultured at 37 °C in Middlebrook 7H9 broth supplemented with 10% oleic acid-albumindextrose-catalase (OADC), 0.5% glycerol, and 0.05% Tween80, or grown on Middlebrook 7H11 agar media supplemented with 10% OADC and 0.5% glycerol. When required, kanamycin, zeocin, and doxycycline were added to the media at final concentrations of 200 µg/mL, 300 µg/mL, and 100 ng/mL, respectively. *Escherichia coli* DH5*α* was used for pGRNAz plasmid construction and cloning, cultured in Lysogeny broth (LB) broth at room temperature or 37 °C, supplemented with 50 µg/mL zeocin when necessary. Bacterial strains used in this study are listed in *SI Appendix*, Table S1.

### Construction of MAB_1132c KO Mutant Strains.

sgRNAs were designed targeting MAB_1132c (listed in *SI Appendix*, Table S2) and cloned into the pGRNAz plasmid upstream of the terminator and *cas9* handle sequence. Recombinant pGRNAz plasmid containing one of the sgRNA pairs was then transformed into *M. abscessus* pTetInt-*cas9*PYO by electroporation. Expression of the sgRNAs and Cas9 was induced by growing the *M. abscessus* strains with 100 ng/mL doxycycline at 37 °C shaking for 48 h. Cultures were then serially diluted and plated onto Middlebrook 7H11 plates containing kanamycin (200 µg/mL) and zeocin (300 µg/mL). Knockout mutations were identified by PCR and Sanger sequencing (primers listed in *SI Appendix*, Table S3).

Three independent ∆MAB_1132c strains were generated. Sanger sequencing was used to confirm that these strains did not carry additional mutations in the closely related MAB_2914 gene.

### Growth Curves.

For growth curves with various amino acids as the sole nitrogen source, *M. abscessus* was grown in Sauton’s Modified Media containing 0.5 g/L monopotassium phosphate, 0.5 g/L magnesium sulfate, 2 g/L citric acid, 10 g/L glycerol, 0.05% Tween80, and 5 mM asparagine, aspartate, glutamine, or glutamate (adjusted to pH 7.0). For growth curves with increasing concentrations of asparagine, *M. abscessus* strains were grown in Sauton’s Modified Media with 5 mM, 10 mM, 20 mM, or 40 mM asparagine. Precultures of each strain were pelleted, washed three times in PBS-Tween80 (0.1%), and diluted to OD_600_ = 1.0. Then, 2 µL of this bacterial preparation was used to inoculate 198 µL of each media in a 96-well plate. Cultures were performed in quadruplicate at 37 °C shaking, and bacterial growth was measured by gently resuspending each culture and measuring OD_592_ in a 96-well plate reader at each time point.

### Nitrogen-Limited Growth.

Precultures of *M. abscessus* strains were growth in Middlebrook 7H9, pelleted, and washed three times in PBS-Tween80 (0.1%). The bacterial suspension was diluted in PBS-Tween80 (0.1%) to OD_600_ = 1.0, and 100 µL of this was used to inoculate 9.9 mL of either Middlebrook 7H9 or Sauton’s Modified Media with 5 mM asparagine. Cultures were then grown at 37 °C shaking for 3 d. To create nitrogen limitation in the wild-type bacteria, cultures were grown in Sauton’s Modified Media with ammonium chloride as the sole nitrogen source, at either 0.05 g/L for low nitrogen or 1 g/L for high nitrogen.

### *Drosophila* Infection, Survival Assays, and Bacterial Load Quantifications.

*Drosophila* stocks were maintained on a standard diet containing 10% w/v Brewer’s yeast, 8% w/v fructose, 2% w/v polenta, 0.8% w/v agar, supplemented with 0.0825% vol propionic acid and 0.075 % w/v nipagin at 25 °C until infection. Male flies between 5 and 8 d old were used for all experiments. w^1118^ flies were used as wild-type flies; all fly lines used in this study are listed in *SI Appendix*, Table S4. Male flies were chosen for all experiments to reduce the frequency of tipping infected flies onto fresh food, as egg-laying females can liquefy the food faster, which leads to flies becoming stuck, interfering with survival analyses.

To deplete insulin-producing cells (IPCs), larvae were allowed to develop at room temperature; then, adult flies were collected 1 d following eclosion and placed at 29 °C for 6 d to kill ilp2-expressing cells by reaper-driven apoptosis.

*M. abscessus* cultures were grown for 3 d, pelleted by centrifugation at 4,000×g for 5 min, and washed in PBSTween80 (0.1%) and diluted in PBS-Tween80 (0.1%) to OD_600_ = 0.05. Flies were anesthetized with CO_2_ and injected with bacteria using a pulled borosilicate glass capillary needle. A picospritzer III system was used to inject 50 nL of bacterial suspension or sterile PBS-Tween80 (0.1%) into the lateral anterior abdomen. Postinfection flies were maintained at 29 °C and 65% humidity and transferred to new vials with fresh food every 3 or 4 d. For survival assays, the number of dead flies was recorded at least twice per day.

For bacterial load quantifications, flies were injected and maintained as above. At each time point, 8 flies were collected and homogenized in 100 µL PBS-Tween80 (1%) and serially diluted in PBS-Tween80 (1%). 10 µL per dilution was plated onto Middlebrook 7H11 agar plates and incubated at 29 °C for 3 d. Colonies were counted to calculate the number of colony-forming units (CFU) per fly.

### RT-qPCR.

For RT-qPCR of bacterial cultures, *M. abscessus* cultures were grown in 10 mL of media for 2 or 3 d, pelleted by centrifugation at 4,000×g for 5 min, and resuspended in 400 µL TRI reagent. They were then transferred into a 2 mL tube containing 500 µL zirconium beads and placed into a bead beater for 2 min, followed by 1 min rest, and then beat for another 2 min. The tubes were centrifuged at 13,000 rpm for 2 min at 4 °C, and the TRI reagent supernatant was recovered in a 1.5 mL microcentrifuge tube. For RT-qPCR of *Drosophila*, 3 flies per condition were collected and homogenized in 100 µL TRI reagent.

RNA was isolated from TRI reagent according to the manufacturer’s instructions and washed in 70% ethanol. The RNA pellet was treated with RNAse-free DNAse I, primed with random hexamers and reverse transcribed using RevertAid M-MuLV reverse transcriptase to produce cDNA. 5 µL of each cDNA sample was pooled and serially diluted to create a standard curve; the remaining cDNA was diluted in TE (10 mM Tris, 1 mM EDTA, pH 7.6) and used for analysis. qPCR was performed using SyGreen 2x qPCR mix on a Corbett Rotor-Gene 6000 with the following cycling conditions: hold at 95 °C for 10 min, 45 cycles of 95 °C for 15 s, 57 °C for 30 s, and 72 °C for 30 s, followed by a melting curve. Gene expression was determined by comparison of each value to the standard curve and normalized to *MAB_3869c* (*rpoB*) for *M. abscessus* gene expression, or *RpL4* for *Drosophila* gene expression. RT-qPCR primers for *M. abscessus* gene expression are listed in *SI Appendix*, Table S5, and primers for *D. melanogaster* gene expression are listed in *SI Appendix*, Table S6.

### Western Blots.

Three flies were collected and homogenized in 75 µL 2 × Laemmli buffer (100 mM Tris pH 6.8, 20% glycerol, 4% SDS, and 0.2 M DTT). Lysate was heated at 85 °C for 5 min; then, 5 µL was loaded into 4 to 12% Bis-Tris gels and run, and proteins were then transferred onto nitrocellulose membranes. Primary antibodies used were anti-phospho-AKT (Cell Signalling Technologies 4054, 1:1,000), anti-total-AKT (Cell Signalling Technologies 4691, 1:1,000), and anti-*α*-Tubulin (Developmental Studies Hybridoma Bank 12G10, 1:5,000). Secondary antibodies were anti-rabbit IgG (Cell Signalling Technologies 7074, 1:5,000), and anti-mouse IgG (Cell Signalling Technologies 7076, 1:10,000). SuperSignal West Pico was used to detect the proteins, and blots were imaged using a Fuji LAS-3000 luminescent image analyzer. Blots were analyzed using ImageJ.

### Triglyceride Measurement.

Thin layer chromatography (TLC) was used to measure bacterial triglycerides. Briefly, bacterial cultures were diluted to a consistent OD, pelleted by centrifugation at 4,000×g for 10 min, and dried. Pellets were then resuspended in 100 µL of a chloroform (3): methanol (1) solution and spun for 3 min at 13,000 rpm at 4 °C. The pellets were homogenized, spun again, and maintained on ice. Standards were produced using lard dissolved in chloroform (3): methanol (1) mix, diluting each standard to known concentrations. For each experiment, at least three replicates of each sample were run. 2 µL of each standard and 20 µL of each sample were loaded onto silica gel glass plates, placed into a chamber containing the solvent hexane (4): ethyl ether (1), and run until the solvent was 1 cm below the top edge of the plate. Plates were removed from the chamber and dried. To visualize triglycerides, the plates were stained with CAM solution, baked at 80 °C for 20 min, and scanned. Plates were analyzed using ImageJ.

### Statistics.

Data were analyzed in R Studio with R version (4.2.3). Survival data were compared using log-rank tests for pairwise comparisons; for all other tests, we first tested for normality to select whether a *t* test, ANOVA, or Kruskal–Wallis test should be used.

## Supplementary Material

Appendix 01 (PDF)

## Data Availability

Miscellaneous data not included in manuscript data have been deposited in Zenodo (10.5281/zenodo.13907667) (49).
