# Supplementary material for: Microbial metabolism disrupts cytokine activity to impact host immune response
Source: Proc Natl Acad Sci U S A. 2024 Nov 8;121(46):e2405719121. doi: 10.1073/pnas.2405719121 (PMC11573640; doi:10.1073/pnas.2405719121)
Supplement: Supplementary file 1 — Appendix 01 (PDF) [file pnas.2405719121.sapp.pdf]

Supporting Information for

Microbial metabolism disrupts cytokine activity to impact host immune response.

Eleanor K. P. Marshall, Catarina Nunes, Sophie Burbaud, Crystal M. Vincent, Natalie O. Munroe, Carolina J. Simoes da Silva, Ashima Wadhawan, William H. Pearson, Jasper Sangen, Lucas Boeck, R. Andres Floto, Marc S. Dionne

Marc S. Dionne.
E-mail: m.dionne@imperial.ac.uk

This PDF file includes:

Figs. S1 to S4
Tables S1 to S8

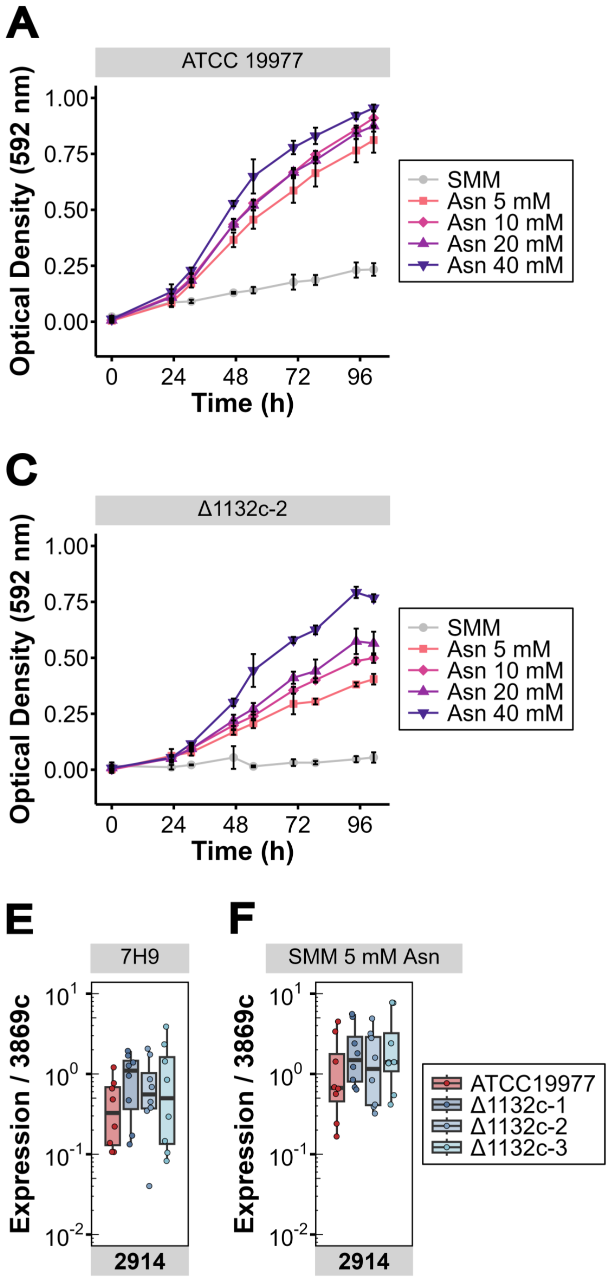

Fig. S1. Increasing asparagine concentrations rescues growth of △MAB_1132c strains Growth of *M. abscessus* (**A**) wild type (ATCC 19977) and (**B, C, D**) △MAB_1132c strains in minimal media with increasing concentrations of asparagine as the sole nitrogen source. SMM (grey) shows growth in minimal media with no nitrogen source provided. Measured by optical density at 592 nm. Data represent the mean ± s.d of quadruplicate samples and are representative of three independent experiments. RT-qPCRs show gene expression of gene MAB_2914, also annotated as an asparagine permease, during exponential phase growth in (**E**) 7H9 complete media or in (**F**) minimal media with 5 mM asparagine as the sole nitrogen source. Data are representative of two independent experiments (N = 8), normalised against MAB_3869c (*rpoB*), and were compared using a Kruskal Wallis ANOVA.

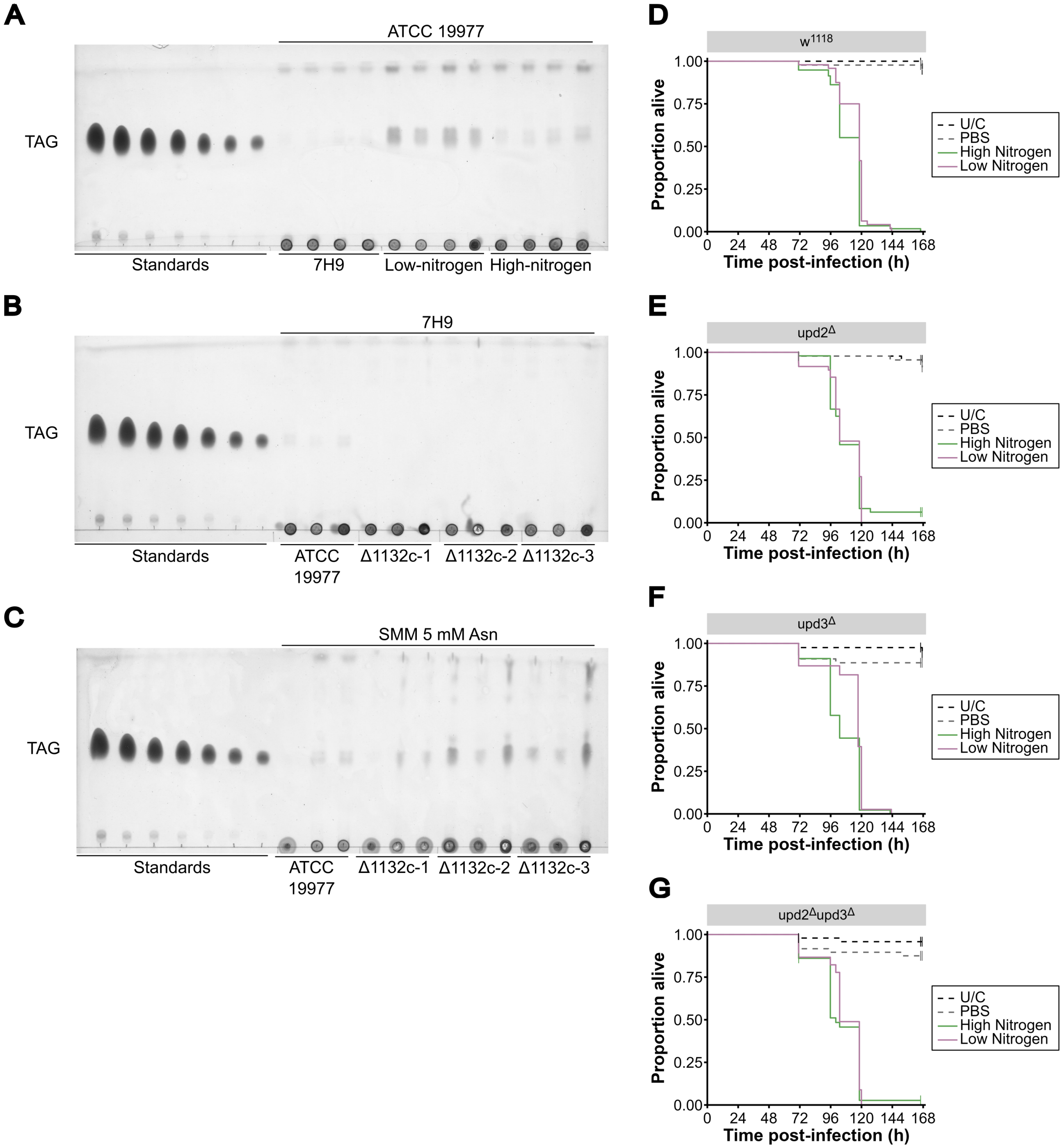

Fig. S2. Nitrogen limitation phenotypes in *M. abscessus* strains. (**A**) Cultures of wild-type (ATCC 19977) *M. abscessus* were grown in 7H9 complete media, or Sauton's minimal media containing either high nitrogen (SMM HN) or low nitrogen (SMM LN) concentrations, triacylglycerols were extracted and TLC plates were run with lard standards. Cultures of wild-type (ATCC 19977) *M. abscessus* and $\triangle$MAB_1132c strains were grown in (**B**) 7H9 complete media or (**C**) SMM 5 mM Asn , triacylglycerols were extracted and TLC plates were run with lard standards. Survival curves of (**D**) w^{1118}, (**E**) upd2$^{\triangle}$, (**F**) upd3$^{\triangle}$, and (**G**) upd2$^{\triangle}$upd3$^{\triangle}$ flies infected with *M. abscessus* ATCC 19977 grown in either Sauton's minimal media containing either high nitrogen (SMM HN) (green) or low nitrogen (SMM LN) (purple) concentrations. Control groups are uninfected flies (black) and PBS Tween80 0.1% injected flies (grey). Data represent two independent experiments with a minimum of 20 flies per group per experiment, and were analysed using Log-Rank test.

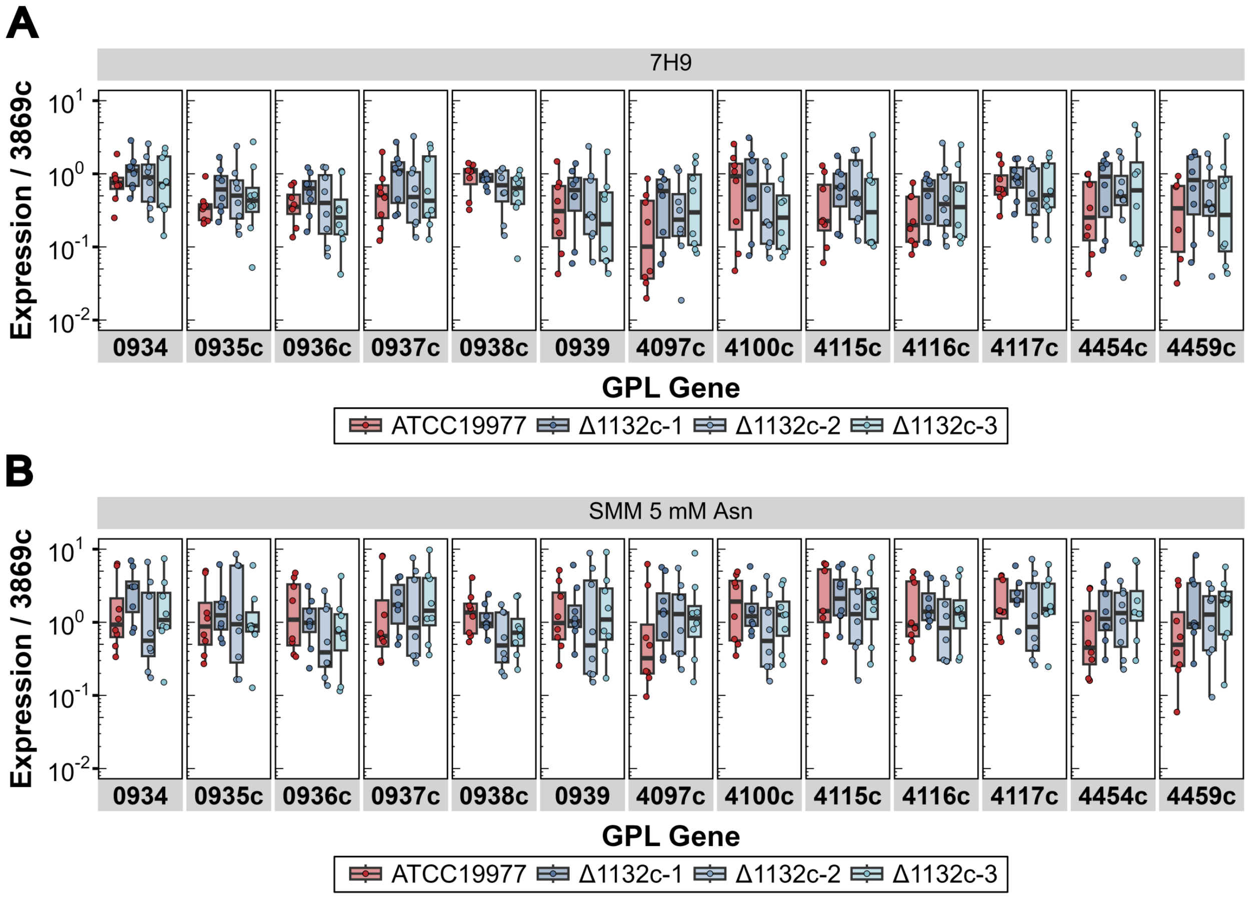

Fig. S3. No changes in glycopeptidolipid gene transcription in △MAB_1132c strains. RT-qPCRs show gene expression of genes involved in glycopeptidolipid (GPL) production in *M. abscessus* wild type (ATCC 19977) and △MAB_1132c strains during exponential phase growth in (**A**) complete 7H9 media or in (**B**) Sauton's minimal media (SMM) with 5 mM asparagine as the sole nitrogen source. Data are representative of two independent experiments (N = 8), normalised against MAB_3869c (*rpoB*), and were compared using a Kruskal Wallis ANOVA. ∗, $P < 0.05$; ∗∗, $P < 0.01$; ∗∗∗, $P < 0.001$; ∗∗∗∗, $P < 0.0001$.

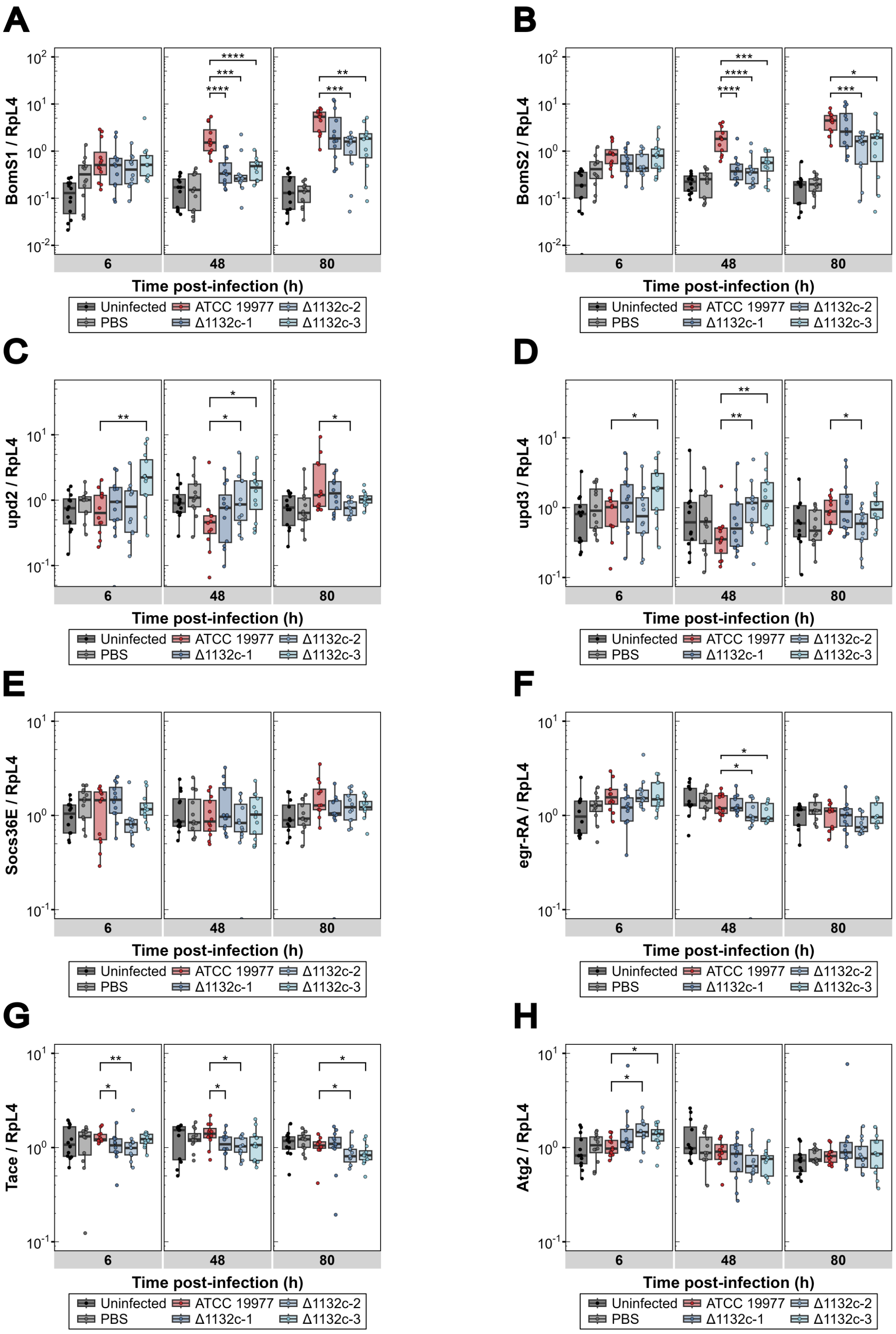

Fig. S4. Transcription of various immune and metabolic genes in *D. melanogaster* infected with *M. abscessus* continued. RT-qPCRs show gene expression of *D. melanogaster* that are uninfected black, injected with PBS as sterile injury control (grey), or infected with *M. abscessus* wild-type (ATCC 19977) (red) or a △MAB_1132c strain (blue) at 6 hours, 48 hours, or 80 hours post-infection. Transcripts measured are (**A**) upd2, (**B**) upd3, (**C**) Socs36E, (**D**) Atg2, (**E**) Egr, (**F**) Tace, (**G**) BomS1, and (**H**) BomS2. Data are representative of three independent experiments (N = 12), normalised against RpL4, and were compared using a Kruskal Wallis ANOVA. Statistical comparisons between *M. abscessus* ATCC 19977 and the △MAB_1132c strains are shown, all statistically significant comparisons are available in Supplementary Table S8. ∗, P < 0.05; ∗∗, P < 0.01; ∗∗∗, P < 0.001; ∗∗∗∗, P < 0.0001.

Strain	Description	Source
ATCC 19977	*Mycobacterium abscessus* type strain	NCTC
ATCC 19977-*cas9*	*Mycobacterium abscessus* type strain transformed with plasmid pTetInt-*cas9*PYO	Andres Floto
ΔMAB_1132c-1	*Mycobacterium abscessus* with frameshift mutation (c.160del) in MAB_1132c	This study
ΔMAB_1132c-2	*Mycobacterium abscessus* with frameshift mutation (c.199-218del) in MAB_1132c	This study
ΔMAB_1132c-3	*Mycobacterium abscessus* with frameshift mutation (c.256-257insG) in MAB_1132c	This study
DH5α	*Escherichia coli* cloning strain	NEB

Table S1. Bacterial strains used in this study. List of bacterial strains used in experiments or to generate CRISPR mutants.

Guide	Forward sgRNA	Reverse sgRNA
1132c g1	ACACCACGAACAGTCCGGGC	CGGCCCGGACTGTTCGTGGTGTCATG
1132c g2	CGCAGCATGAAGAAGACGAA	CGTTCGTCTTCTTCATGCTGCGCATG
1132c g3	GAGCATAGGAGACGAAGGAG	CGCTCCTTCGTCTCCTATGCTCCATG

Table S2. *Mycobacterium abscessus* CRISPR single guide RNAs.

Gene	Forward	Reverse
MAB_1132c	TGGTCAACACGATGATCAGG	CGTGGCCAATCTCATACCG
MAB_2914	CGATGGACCACTGAGGGATG	GCCGATCATTTGGACTTGCC

Table S3. *Mycobacterium abscessus* PCR primers used in this study. Primers used to validate CRISPR-induced mutations in *M. abscessus*.

Line	Description	Source
ilp2-Gal4	Insulin producing cell specific Gal4 driver line	Dionne laboratory stocks
imd^{10191}	Loss of function allele of *imd*	Dionne laboratory stocks
spz$^{\Delta 8-1}$	350 bp deletion including the *spz* start codon	Shoichiro Kurata, Tohoku University
tub-Gal80$^{[ts]}$*; UAS-reaper*	Expression of pro-apoptotic gene *reaper* under the control of UAS, with temperature sensitive inhibition	Dionne laboratory stocks
upd2$^{\Delta}$	4.7 kb deletion removing the *upd2* transcription unit	B. Lemaitre, EPFL
upd3$^{\Delta}$	13,464 bp deletion removing the first three exons of *upd3*	B. Lemaitre, EPFL
upd2$^{\Delta}$ *upd3*$^{\Delta}$	Double mutant containing deletion alleles of *upd2* and *upd3*	B. Lemaitre, EPFL
w^{1118}	Isogenic wild-type control	BDSC

Table S4. *Drosophila melanogaster* lines used in this study.

Gene	Forward	Reverse
MAB_0934	ATTCCTGTCCGGTGGGTTTG	ATCCGGTCGATACATTGCGA
MAB_0935c	GGATCATCGACAGCGACACT	CGGGGGACTTACGCCAATAG
MAB_0936c	GCAACATCGTGACCGAAACC	CACCCCGAGGTACTTGAAGC
MAB_0937c	CGGACTATGCCGTCTTCCTC	CTGACGGCTTGGTCGTAGTT
MAB_0938c	CGGTTCTCCTACCAGCTGAC	CTGATTTCTCGGCGATCGGA
MAB_0939	CATGAGAGCCCGCTGGATAG	GCACGTTTGACGGTGAACTC
MAB_2914	ATTGGTCATCGTCACCTCGG	TGTCAGGGTTTTCGGTCTCG
MAB_3869c	TGATCAACATCCGTCCCGTC	CTGGTCCATGAACTGCGACA
MAB_4097c	GTCGTCATAGCCGTGTGGAT	GTCCAGTACCAAGACCGACG
MAB_4100c	CCTGCCTGGAGTACATCGAA	CCACCTTGCGCCAATCTCTC
MAB_4115c	GATGAACGGTCGGTATCGCT	TGTCCGAGTTCGACTCGTTG
MAB_4116c	AGCGAGAGCATTCGGTTTCA	AAAGACCTTCCCCATCTGCG
MAB_4117c	GATTCCGCTGCTGGTGATTG	TCTCGTTACCGAATACGCCG
MAB_4454c	TTCTGGATCATCAACGCCCC	CACCTCTACGGCCTCTTCAC
MAB_4459c	GAGCCGATCTCGACCGTTAC	TTCTCGGTCAGTTCCAAGGC

Table S5. *Mycobacterium abscessus* RT-qPCR primers used in this study.

Gene	Forward	Reverse
Atg2	CAAAAGACCTGTACGCAGAGC	TACTGAGCAACAGGTTTCGTC
AttA	CACAATGTGGTGGGTCAGG	GGCACCATGACCAGCATT
BomS1	CGTCACCGTTTTTGTGCTC	TGATCACATTTCCTGGATCG
BomS2	ACCGTCTTTGTGTTCGGTCT	GATTACCACATTTCCTGGATCG
Def	TTCTCGTGGCTATCGCTTTT	GGAGAGTAGGTCGCATGTGG
Dipt	ACCGCAGTACCCACTCAATC	CCCAAGTGCTGTCCATATCC
Dro	CCATCGAGGATCACCTGACT	CTTTAGGCGGGCAGAATG
Drs	GTACTTGTTCGCCCTCTTCG	CTTGCACACACGACGACAG
egr-RA	GAGGCAACTTCCAAAGAGAGC	CGGATCTGGCTGAAAGAAGA
Mtk	TCTTGGAGCGATTTTTCTGG	TCTGCCAGCACTGATGTAGC
PepCK	GGATAAGGTGGACGTGAAGG	ACCTCCTGCGACCAGAACT
RpL4	TCCACCTTGAAGAAGGGCTA	TTGCGGATCTCCTCAGACTT
Socs36E	AAAAAGCCAGCAAACCAAAA	AGGTGATGACCCATTGGAAG
Tace	CTGATGTCCTACCCGATGGC	CCTCAAGGTCTTGGCCACAT
Thor	CAGATGCCCGAGGTGTACTC	CATGAAAGCCCGCTCGTAGA
TotA	CCAAAATGAATTCTTCAACTGC	GAATAGCCCATGCATAGAGGAC
upd2	CGGAACATCACGATGAGCGAAT	TCGGCAGGAACTTGTACTCG
upd3	ACTGGGAGAACACCTGCAAT	GCCCGTTTGGTTCTGTAGAT

Table S6. *Drosophila melanogaster* RT-qPCR primers used in this study.

Table S7. Statistically significant comparisons for Figure ??.

Gene	Time	Comparison	P-value
Drs	6 hr	UI vs PBS	0.5 (*)
Drs	6 hr	UI vs ATCC19977	0.1 (**)
Drs	6 hr	UI vs ΔMAB_1132c-1	0.31 (*)
Drs	6 hr	UI vs ΔMAB_1132c-2	0.5 (*)
Drs	6 hr	UI vs ΔMAB_1132c-3	0.017 (**)
Drs	48 hr	UI vs ATCC19977	0.00021 (****)
Drs	48 hr	PBS vs ATCC19977	0.013 (***)
Drs	48 hr	ATCC19977 vs ΔMAB_1132c-1	0.017 (**)
Drs	48 hr	ATCC19977 vs ΔMAB_1132c-2	0.000044 (****)
Drs	48 hr	ATCC19977 vs ΔMAB_1132c-3	0.021 (**)
Drs	80 hr	UI vs ATCC19977	0.000011 (****)
Drs	80 hr	UI vs ΔMAB_1132c-1	0.0015 (***)
Drs	80 hr	UI vs ΔMAB_1132c-2	0.1 (**)
Drs	80 hr	UI vs ΔMAB_1132c-3	0.0098 (***)
Drs	80 hr	PBS vs ATCC19977	0.000011 (****)
Drs	80 hr	PBS vs ΔMAB_1132c-1	0.0015 (***)
Drs	80 hr	PBS vs ΔMAB_1132c-2	0.083 (**)
Drs	80 hr	PBS vs ΔMAB_1132c-3	0.027 (**)
Drs	80 hr	ATCC19977 vs ΔMAB_1132c-1	0.035 (**)
Drs	80 hr	ATCC19977 vs ΔMAB_1132c-2	0.0015 (***)
Drs	80 hr	ATCC19977 vs ΔMAB_1132c-3	0.003 (***)
Mtk	6 hr	UI vs PBS	0.000078 (****)
Mtk	6 hr	UI vs ATCC19977	0.000078 (****)
Mtk	6 hr	UI vs ΔMAB_1132c-1	0.000022 (****)
Mtk	6 hr	UI vs ΔMAB_1132c-2	0.000022 (****)
Mtk	6 hr	UI vs ΔMAB_1132c-3	0.000011 (****)
Mtk	48 hr	UI vs ATCC19977	0.000011 (****)
Mtk	48 hr	UI vs ΔMAB_1132c-1	0.021 (**)
Mtk	48 hr	UI vs ΔMAB_1132c-2	0.12 (**)
Mtk	48 hr	UI vs ΔMAB_1132c-3	0.0041 (***)
Mtk	48 hr	PBS vs ATCC19977	0.044 (**)
Mtk	48 hr	ATCC19977 vs ΔMAB_1132c-1	0.0011 (****)
Mtk	48 hr	ATCC19977 vs ΔMAB_1132c-2	0.003 (***)
Mtk	48 hr	ATCC19977 vs ΔMAB_1132c-3	0.027 (**)
Mtk	80 hr	UI vs ATCC19977	0.000011 (****)
Mtk	80 hr	UI vs ΔMAB_1132c-1	0.0011 (****)
Mtk	80 hr	UI vs ΔMAB_1132c-2	0.0022 (***)
Mtk	80 hr	UI vs ΔMAB_1132c-3	0.013 (***)
Mtk	80 hr	PBS vs ATCC19977	0.000011 (****)
Mtk	80 hr	PBS vs ΔMAB_1132c-1	0.00033 (****)
Mtk	80 hr	PBS vs ΔMAB_1132c-2	0.0011 (****)
Mtk	80 hr	PBS vs ΔMAB_1132c-3	0.0056 (***)
Mtk	80 hr	ATCC19977 vs ΔMAB_1132c-2	0.000078 (****)
Mtk	80 hr	ATCC19977 vs ΔMAB_1132c-3	0.00021 (****)
AttA	6 hr	UI vs PBS	0.00013 (****)
AttA	6 hr	UI vs ATCC19977	0.000022 (****)
AttA	6 hr	UI vs ΔMAB_1132c-1	0.000078 (****)
AttA	6 hr	UI vs ΔMAB_1132c-2	0.000011 (****)
AttA	6 hr	UI vs ΔMAB_1132c-3	0.000011 (****)
AttA	48 hr	UI vs ATCC19977	0.000011 (****)
AttA	48 hr	UI vs ΔMAB_1132c-1	0.027 (**)
AttA	48 hr	UI vs ΔMAB_1132c-2	0.31 (*)
AttA	48 hr	UI vs ΔMAB_1132c-3	0.0022 (***)
AttA	48 hr	PBS vs ATCC19977	0.0022 (***)
AttA	48 hr	PBS vs ΔMAB_1132c-3	0.36 (*)
AttA	48 hr	ATCC19977 vs ΔMAB_1132c-1	0.000011 (****)

Gene	Time	Comparison	P-value
AttA	48 hr	ATCC19977 vs ΔMAB_1132c-2	0.003 (***)
AttA	48 hr	ATCC19977 vs ΔMAB_1132c-3	0.044 (**)
AttA	80 hr	UI vs ATCC19977	0.000011 (****)
AttA	80 hr	UI vs ΔMAB_1132c-1	0.00074 (****)
AttA	80 hr	UI vs ΔMAB_1132c-2	0.013 (***)
AttA	80 hr	UI vs ΔMAB_1132c-3	0.0015 (***)
AttA	80 hr	PBS vs ATCC19977	0.000011 (****)
AttA	80 hr	PBS vs ΔMAB_1132c-1	0.0005 (****)
AttA	80 hr	PBS vs ΔMAB_1132c-2	0.021 (**)
AttA	80 hr	PBS vs ΔMAB_1132c-3	0.0022 (***)
AttA	80 hr	ATCC19977 vs ΔMAB_1132c-1	0.000078 (****)
AttA	80 hr	ATCC19977 vs ΔMAB_1132c-2	0.000078 (****)
AttA	80 hr	ATCC19977 vs ΔMAB_1132c-3	0.00074 (****)
Dipt	6 hr	UI vs PBS	0.000044 (****)
Dipt	6 hr	UI vs ATCC19977	0.000044 (****)
Dipt	6 hr	UI vs ΔMAB_1132c-1	0.00013 (****)
Dipt	6 hr	UI vs ΔMAB_1132c-2	0.000011 (****)
Dipt	6 hr	UI vs ΔMAB_1132c-3	0.000011 (****)
Dipt	6 hr	ΔMAB_1132c-1 vs ΔMAB_1132c-3	0.58 (*)
Dipt	48 hr	UI vs ATCC19977	0.0011 (****)
Dipt	48 hr	UI vs ΔMAB_1132c-3	0.36 (*)
Dipt	48 hr	PBS vs ATCC19977	0.36 (*)
Dipt	48 hr	ATCC19977 vs ΔMAB_1132c-1	0.0098 (***)
Dipt	48 hr	ATCC19977 vs ΔMAB_1132c-2	0.021 (**)
Dipt	80 hr	UI vs ATCC19977	0.000011 (****)
Dipt	80 hr	UI vs ΔMAB_1132c-1	0.5 (*)
Dipt	80 hr	UI vs ΔMAB_1132c-2	0.1 (**)
Dipt	80 hr	UI vs ΔMAB_1132c-3	0.055 (**)
Dipt	80 hr	PBS vs ATCC19977	0.000011 (****)
Dipt	80 hr	PBS vs ΔMAB_1132c-1	0.58 (*)
Dipt	80 hr	PBS vs ΔMAB_1132c-2	0.22 (*)
Dipt	80 hr	PBS vs ΔMAB_1132c-3	0.12 (**)
Dipt	80 hr	ATCC19977 vs ΔMAB_1132c-1	0.0011 (****)
Dipt	80 hr	ATCC19977 vs ΔMAB_1132c-2	0.0056 (***)
Dipt	80 hr	ATCC19977 vs ΔMAB_1132c-3	0.027 (**)
Dro	6 hr	UI vs PBS	0.00013 (****)
Dro	6 hr	UI vs ATCC19977	0.00013 (****)
Dro	6 hr	UI vs ΔMAB_1132c-1	0.00033 (****)
Dro	6 hr	UI vs ΔMAB_1132c-2	0.00033 (****)
Dro	6 hr	UI vs ΔMAB_1132c-3	0.00013 (****)
Dro	48 hr	UI vs PBS	0.18 (*)
Dro	48 hr	UI vs ATCC19977	0.000022 (****)
Dro	48 hr	UI vs ΔMAB_1132c-1	0.0098 (***)
Dro	48 hr	UI vs ΔMAB_1132c-2	0.021 (**)
Dro	48 hr	UI vs ΔMAB_1132c-3	0.00074 (****)
Dro	48 hr	PBS vs ATCC19977	0.67 (*)
Dro	48 hr	ATCC19977 vs ΔMAB_1132c-1	0.027 (**)
Dro	48 hr	ATCC19977 vs ΔMAB_1132c-2	0.0022 (***)
Dro	48 hr	ATCC19977 vs ΔMAB_1132c-3	0.58 (*)
Dro	80 hr	UI vs ATCC19977	0.000011 (****)
Dro	80 hr	UI vs ΔMAB_1132c-1	0.0022 (***)
Dro	80 hr	UI vs ΔMAB_1132c-2	0.0098 (***)
Dro	80 hr	UI vs ΔMAB_1132c-3	0.0074 (***)
Dro	80 hr	PBS vs ATCC19977	0.000011 (****)
Dro	80 hr	PBS vs ΔMAB_1132c-1	0.0056 (***)
Dro	80 hr	PBS vs ΔMAB_1132c-2	0.035 (**)
Dro	80 hr	PBS vs ΔMAB_1132c-3	0.013 (***)
Dro	80 hr	ATCC19977 vs ΔMAB_1132c-1	0.15 (*)

Gene	Time	Comparison	P-value
Dro	80 hr	ATCC19977 vs ΔMAB_1132c-2	0.000011 (****)
Dro	80 hr	ATCC19977 vs ΔMAB_1132c-3	0.000022 (****)
TotA	6 hr	UI vs ΔMAB_1132c-2	0.0015 (***)
TotA	6 hr	PBS vs ΔMAB_1132c-2	0.044 (**)
TotA	6 hr	ATCC19977 vs ΔMAB_1132c-2	0.15 (*)
TotA	6 hr	ΔMAB_1132c-1 vs ΔMAB_1132c-2	0.31 (*)
TotA	48 hr	PBS vs ΔMAB_1132c-2	0.43 (*)
TotA	48 hr	PBS vs ΔMAB_1132c-3	0.43 (*)
TotA	80 hr	UI vs PBS	0.083 (**)
TotA	80 hr	UI vs ATCC19977	0.0098 (***)
TotA	80 hr	UI vs ΔMAB_1132c-2	0.5 (*)
TotA	80 hr	PBS vs ATCC19977	0.000011 (****)
TotA	80 hr	PBS vs ΔMAB_1132c-2	0.000044 (****)
TotA	80 hr	PBS vs ΔMAB_1132c-3	0.0005 (****)
TotA	80 hr	ATCC19977 vs ΔMAB_1132c-1	0.0041 (***)
TotA	80 hr	ATCC19977 vs ΔMAB_1132c-2	0.43 (*)
TotA	80 hr	ATCC19977 vs ΔMAB_1132c-3	0.36 (*)
TotA	80 hr	ΔMAB_1132c-1 vs ΔMAB_1132c-2	0.22 (*)
TotA	80 hr	ΔMAB_1132c-1 vs ΔMAB_1132c-3	0.67 (*)

Table S8. Statistically significant comparisons for Supplementary Figure S4.

Gene	Time	Comparison	P-value
upd2	6 hr	UI vs ΔMAB_1132c-3	0.068 (∗∗)
upd2	6 hr	PBS vs ΔMAB_1132c-3	0.1 (∗∗)
upd2	6 hr	ATCC19977 vs ΔMAB_1132c-3	0.055 (∗∗)
upd2	6 hr	ΔMAB_1132c-1 vs ΔMAB_1132c-3	0.5 (∗)
upd2	6 hr	ΔMAB_1132c-2 vs ΔMAB_1132c-3	0.31 (∗)
upd2	48 hr	UI vs ATCC19977	0.055 (∗∗)
upd2	48 hr	PBS vs ATCC19977	0.055 (∗∗)
upd2	48 hr	ATCC19977 vs ΔMAB_1132c-2	0.67 (∗)
upd2	48 hr	ATCC19977 vs ΔMAB_1132c-3	0.26 (∗)
upd2	80 hr	PBS vs ATCC19977	0.26 (∗)
upd2	80 hr	ATCC19977 vs ΔMAB_1132c-2	0.18 (∗)
upd2	80 hr	ΔMAB_1132c-1 vs ΔMAB_1132c-2	0.58 (∗)
upd2	80 hr	ΔMAB_1132c-2 vs ΔMAB_1132c-3	0.26 (∗)
upd3	6 hr	UI vs ΔMAB_1132c-3	0.31 (∗)
upd3	6 hr	ATCC19977 vs ΔMAB_1132c-3	0.26 (∗)
upd3	6 hr	ΔMAB_1132c-2 vs ΔMAB_1132c-3	0.67 (∗)
upd3	48 hr	ATCC19977 vs ΔMAB_1132c-2	0.055 (∗∗)
upd3	48 hr	ATCC19977 vs ΔMAB_1132c-3	0.017 (∗∗)
upd3	80 hr	PBS vs ATCC19977	0.58 (∗)
upd3	80 hr	PBS vs ΔMAB_1132c-1	0.5 (∗)
upd3	80 hr	PBS vs ΔMAB_1132c-3	0.22 (∗)
upd3	80 hr	ATCC19977 vs ΔMAB_1132c-2	0.58 (∗)
upd3	80 hr	ΔMAB_1132c-2 vs ΔMAB_1132c-3	0.58 (∗)
Socs36E	6 hr	UI vs ΔMAB_1132c-1	0.58 (∗)
Socs36E	6 hr	PBS vs ΔMAB_1132c-2	0.18 (∗)
Socs36E	6 hr	ΔMAB_1132c-1 vs ΔMAB_1132c-2	0.068 (∗∗)
Socs36E	6 hr	ΔMAB_1132c-2 vs ΔMAB_1132c-3	0.044 (∗∗)
Socs36E	80 hr	UI vs ATCC19977	0.31 (∗)
Socs36E	80 hr	PBS vs ATCC19977	0.43 (∗)
Atg2	6 hr	UI vs ΔMAB_1132c-1	0.43 (∗)
Atg2	6 hr	UI vs ΔMAB_1132c-2	0.12 (∗∗)
Atg2	6 hr	UI vs ΔMAB_1132c-3	0.58 (∗)
Atg2	6 hr	PBS vs ΔMAB_1132c-2	0.18 (∗)
Atg2	6 hr	ATCC19977 vs ΔMAB_1132c-2	0.18 (∗)
Atg2	6 hr	ATCC19977 vs ΔMAB_1132c-3	0.31 (∗)
Atg2	48 hr	UI vs ΔMAB_1132c-2	0.5 (∗)
Atg2	48 hr	UI vs ΔMAB_1132c-3	0.068 (∗∗)
Atg2	80 hr	UI vs ΔMAB_1132c-1	0.31 (∗)
egr-RA	6 hr	UI vs ATCC19977	0.26 (∗)
egr-RA	6 hr	UI vs ΔMAB_1132c-2	0.068 (∗∗)
egr-RA	6 hr	UI vs ΔMAB_1132c-3	0.31 (∗)
egr-RA	6 hr	PBS vs ΔMAB_1132c-2	0.5 (∗)
egr-RA	48 hr	UI vs ΔMAB_1132c-2	0.43 (∗)
egr-RA	48 hr	PBS vs ΔMAB_1132c-2	0.22 (∗)
egr-RA	48 hr	PBS vs ΔMAB_1132c-3	0.083 (∗∗)
egr-RA	48 hr	ATCC19977 vs ΔMAB_1132c-2	0.5 (∗)
egr-RA	48 hr	ATCC19977 vs ΔMAB_1132c-3	0.58 (∗)
egr-RA	48 hr	ΔMAB_1132c-1 vs ΔMAB_1132c-2	0.58 (∗)
egr-RA	80 hr	UI vs ΔMAB_1132c-2	0.5 (∗)
egr-RA	80 hr	PBS vs ΔMAB_1132c-2	0.12 (∗∗)
egr-RA	80 hr	ΔMAB_1132c-2 vs ΔMAB_1132c-3	0.43 (∗)
Tace	6 hr	ATCC19977 vs ΔMAB_1132c-1	0.58 (∗)
Tace	6 hr	ATCC19977 vs ΔMAB_1132c-2	0.035 (∗∗)
Tace	6 hr	ΔMAB_1132c-2 vs ΔMAB_1132c-3	0.36 (∗)
Tace	48 hr	ATCC19977 vs ΔMAB_1132c-1	0.58 (∗)
Tace	48 hr	ATCC19977 vs ΔMAB_1132c-2	0.67 (∗)

Gene	Time	Comparison	P-value
Tace	80 hr	UI vs ΔMAB_1132c-2	0.26 (∗)
Tace	80 hr	UI vs ΔMAB_1132c-3	0.26 (∗)
Tace	80 hr	PBS vs ΔMAB_1132c-2	0.055 (∗∗)
Tace	80 hr	PBS vs ΔMAB_1132c-3	0.083 (∗∗)
Tace	80 hr	ATCC19977 vs ΔMAB_1132c-2	0.26 (∗)
Tace	80 hr	ATCC19977 vs ΔMAB_1132c-3	0.58 (∗)
Tace	80 hr	ΔMAB_1132c-1 vs ΔMAB_1132c-2	0.31 (∗)
BomS1	6 hr	UI vs PBS	0.31 (∗)
BomS1	6 hr	UI vs ATCC19977	0.0056 (∗∗∗)
BomS1	6 hr	UI vs ΔMAB_1132c-1	0.055 (∗∗)
BomS1	6 hr	UI vs ΔMAB_1132c-2	0.044 (∗∗)
BomS1	6 hr	UI vs ΔMAB_1132c-3	0.00013 (∗∗∗∗)
BomS1	48 hr	UI vs ATCC19977	0.000011 (∗∗∗∗)
BomS1	48 hr	UI vs ΔMAB_1132c-1	0.26 (∗)
BomS1	48 hr	UI vs ΔMAB_1132c-3	0.013 (∗∗∗)
BomS1	48 hr	PBS vs ATCC19977	0.000011 (∗∗∗∗)
BomS1	48 hr	PBS vs ΔMAB_1132c-1	0.5 (∗)
BomS1	48 hr	PBS vs ΔMAB_1132c-3	0.044 (∗∗)
BomS1	48 hr	ATCC19977 vs ΔMAB_1132c-1	0.0011 (∗∗∗∗)
BomS1	48 hr	ATCC19977 vs ΔMAB_1132c-2	0.003 (∗∗∗)
BomS1	48 hr	ATCC19977 vs ΔMAB_1132c-3	0.00021 (∗∗∗∗)
BomS1	80 hr	UI vs ATCC19977	0.000011 (∗∗∗∗)
BomS1	80 hr	UI vs ΔMAB_1132c-1	0.000044 (∗∗∗∗)
BomS1	80 hr	UI vs ΔMAB_1132c-2	0.013 (∗∗∗)
BomS1	80 hr	UI vs ΔMAB_1132c-3	0.003 (∗∗∗)
BomS1	80 hr	PBS vs ATCC19977	0.000011 (∗∗∗∗)
BomS1	80 hr	PBS vs ΔMAB_1132c-1	0.000011 (∗∗∗∗)
BomS1	80 hr	PBS vs ΔMAB_1132c-2	0.017 (∗∗)
BomS1	80 hr	PBS vs ΔMAB_1132c-3	0.0041 (∗∗∗)
BomS1	80 hr	ATCC19977 vs ΔMAB_1132c-2	0.0015 (∗∗∗)
BomS1	80 hr	ATCC19977 vs ΔMAB_1132c-3	0.021 (∗∗)
BomS2	6 hr	UI vs PBS	0.5 (∗)
BomS2	6 hr	UI vs ATCC19977	0.00021 (∗∗∗∗)
BomS2	6 hr	UI vs ΔMAB_1132c-1	0.013 (∗∗∗)
BomS2	6 hr	UI vs ΔMAB_1132c-2	0.017 (∗∗)
BomS2	6 hr	UI vs ΔMAB_1132c-3	0.0022 (∗∗∗)
BomS2	6 hr	PBS vs ATCC19977	0.26 (∗)
BomS2	48 hr	UI vs ATCC19977	0.000011 (∗∗∗∗)
BomS2	48 hr	UI vs ΔMAB_1132c-1	0.22 (∗)
BomS2	48 hr	UI vs ΔMAB_1132c-2	0.43 (∗)
BomS2	48 hr	UI vs ΔMAB_1132c-3	0.035 (∗∗)
BomS2	48 hr	PBS vs ATCC19977	0.000011 (∗∗∗∗)
BomS2	48 hr	PBS vs ΔMAB_1132c-3	0.055 (∗∗)
BomS2	48 hr	ATCC19977 vs ΔMAB_1132c-1	0.0005 (∗∗∗∗)
BomS2	48 hr	ATCC19977 vs ΔMAB_1132c-2	0.00074 (∗∗∗∗)
BomS2	48 hr	ATCC19977 vs ΔMAB_1132c-3	0.0022 (∗∗∗)
BomS2	80 hr	UI vs ATCC19977	0.000011 (∗∗∗∗)
BomS2	80 hr	UI vs ΔMAB_1132c-1	0.000011 (∗∗∗∗)
BomS2	80 hr	UI vs ΔMAB_1132c-2	0.017 (∗∗)
BomS2	80 hr	UI vs ΔMAB_1132c-3	0.021 (∗∗)
BomS2	80 hr	PBS vs ATCC19977	0.000011 (∗∗∗∗)
BomS2	80 hr	PBS vs ΔMAB_1132c-1	0.000011 (∗∗∗∗)
BomS2	80 hr	PBS vs ΔMAB_1132c-2	0.027 (∗∗)
BomS2	80 hr	PBS vs ΔMAB_1132c-3	0.068 (∗∗)
BomS2	80 hr	ATCC19977 vs ΔMAB_1132c-2	0.0022 (∗∗∗)
BomS2	80 hr	ATCC19977 vs ΔMAB_1132c-3	0.22 (∗)
